# Annotated checklist of freshwater molluscs from the largest freshwater lake in Southeast Asia

**DOI:** 10.3897/zookeys.958.53865

**Published:** 2020-08-11

**Authors:** Ting Hui Ng, Ekgachai Jeratthitikul, Chirasak Sutcharit, Samol Chhuoy, Kakada Pin, Arthit Pholyotha, Warut Siriwut, Ruttapon Srisonchai, Zeb S. Hogan, Peng Bun Ngor

**Affiliations:** 1 Lee Kong Chian Natural History Museum, Faculty of Science, National University of Singapore, 2 Conservatory Drive, 117377, Singapore; 2 Animal Systematics and Molecular Ecology Laboratory, Department of Biology, Faculty of Science, Mahidol University, Bangkok 10400, Thailand; 3 Animal Systematics Research Unit, Department of Biology, Faculty of Science, Chulalongkorn University, 254 Phayathai Road, Pathumwan, Bangkok 10330, Thailand; 4 Inland Fisheries Research and Development Institute (IFReDI), Fisheries Administration, No. 86, Norodom Blvd., PO Box 582, Phnom Penh, Cambodia; 5 Wonders of the Mekong Project, c/o IFReDI, No. 86, Norodom Blvd., PO Box 582, Phnom Penh, Cambodia; 6 Department of Biology, Faculty of Science, Khon Kaen University, Khon Kaen 40002, Thailand; 7 Department of Biology, University of Nevada, 1664 N. Virginia Street, Reno, NV 89557, USA

**Keywords:** alien species, bivalves, Cambodia, diversity, economic species, Lower Mekong basin, snails, Tonle Sap Lake

## Abstract

The Tonle Sap Lake in Cambodia is a crucial freshwater biodiversity hotspot and supports one of the world’s largest inland fisheries. Within the Tonle Sap basin, freshwater molluscs provide vital ecosystem services and are among the fauna targetted for commercial harvesting. Despite their importance, freshwater molluscs of the Tonle Sap basin remain poorly studied. The historical literature was reviewed and at least 153 species of freshwater molluscs have been previously recorded from throughout Cambodia, including 33 from the Tonle Sap basin. Surveys of the Tonle Sap Lake and surrounding watershed were also conducted and found 31 species, 15 bivalves (five families) and 16 gastropods (eight families), in the Tonle Sap basin, including three new records for Cambodia (*Scaphula minuta*, *Novaculina siamensis*, *Wattebledia siamensis*), the presence of globally invasive *Pomacea maculata* and potential pest species like *Limnoperna fortunei*. This study represents the most comprehensive documentation of freshwater molluscs of the Tonle Sap basin, and voucher specimens deposited at the Inland Fisheries Research and Development Institute, Cambodia, represent the first known reference collection of freshwater molluscs in the country. In order to combat the combined anthropogenic pressures, including invasive species, climate change and dams along the Mekong River, a multi-pronged approach is urgently required to study the biodiversity, ecology, ecosystem functioning of freshwater molluscs and other aquatic fauna in the Tonle Sap basin.

## Introduction

“Clams, cockles, snails, et cetera can be obtained just by cupping one’s hands into the Fresh Water Lake” (Zhou in Uk and Uk 2016).

Being the largest natural lake in Southeast Asia, the Tonle Sap Lake is a crucial freshwater habitat for various animals, including fish and birds (Campbell et al. 2006), but besides the above mentioned brief account by the Chinese diplomat, Zhou Daguan, in the 13^th^ century (Uk and Uk 2016), little else is known about the freshwater molluscs of the lake. Although poorly studied, freshwater molluscs in the Tonle Sap basin, as with the fauna in the surrounding Indo-Burmese region, occupy a range of habitats and perform vital ecological roles, interacting with other fauna (Köhler et al. 2012). For its size, Cambodia is the most speciose country in East and Southeast Asia for unionid bivalves; although the data on bivalves in the country requires updating (Zieritz et al. 2018). The most comprehensive checklist of freshwater molluscs for Cambodia was based on material collected almost half a century ago from the main stem of the Mekong River, and did not include the Tonle Sap ecosystem (Brandt and Temcharoen 1971). Recent surveys and ecological studies in the fresh waters of Cambodia have targeted various taxa, and when molluscs were included, all were, at most, identified only to genera (Vongsombath et al. 2009; Ohtaka et al. 2011; Sor et al. 2017).

In addition, there has been interest in economically-important molluscs, including freshwater apple snails (Ampullariidae) and Asian clams (Cyrenidae) (Ngor et al. 2014, 2016, 2018d). The composition of ampullariids in the Tonle Sap Lake appears to vary seasonally, probably owing to the vastly different climatic and hydrological conditions (Ngor et al. 2018a, d). There is also growing concern about the economic and ecological impacts of invasive apple snails, *Pomacea* species, which were not established in Cambodia two decades ago (Cowie 1995), but are now spreading rapidly, including in the Tonle Sap Lake (Ngor et al. 2014; Khay et al. 2018; Ngor PB, pers. obs.). The threat of invasive species, in addition to climate change, flow modification within the Mekong River basin, overharvesting, pollution, and land use change, would most probably impact the ecosystem and biodiversity of the Tonle Sap Lake (Arias et al. 2014, 2019; McCann et al. 2016; Ngor et al. 2018a, b, d; Uk et al. 2018).

Furthermore, most of the recent studies did not include voucher specimens nor photographs of species that could be used by local stakeholders and research scientists. The lack of proper reference material may lead to incorrect identification of species, allowing non-native species to establish and spread unnoticed (e.g., Ng et al. 2015, 2018).

It is therefore imperative to document the biodiversity of freshwater molluscs of Tonle Sap as a foundation for further evolutionary and ecological research, and to make available the necessary information to the government, local residents, and other stakeholders to allow for more effective management of threatened, economic- and medically-important freshwater molluscs in this unique freshwater habitat. In this study, we aimed to conduct 1) a review of historical literature on freshwater molluscs of Cambodia; and 2) surveys of Tonle Sap Lake and the surrounding watershed to provide an updated checklist of species diversity for the freshwater molluscs in the largest lake in Southeast Asia, which is also one of the world’s most productive lakes.

## Materials and methods

### Historical information

Historical data on freshwater mollusc records from Cambodia were gathered from relevant literature based on a search of the Global Biodiversity Information Facility (GBIF.org 2019), the IUCN Red List (IUCN 2018), MolluscaBase (2019), MUSSEL Project (Graf and Cummings 2019), Google and Web of Science using keywords (Cambodia, Cambodge, freshwater, fluviatiles, mollusc, mollusk, mollusques, clam, bivalve, snail, gastropod) or applying relevant filters. Major references included Mabille and Le Mesle (1866), Crosse and Fischer (1876), Morlet (1889), Fischer and Dautzenberg (1904), Brandt and Temcharoen (1971), and Brandt (1974). Only original descriptions and key references, including first record in Cambodia, if applicable, Brandt (1974), and major revisionary work, are listed in the annotated species checklist. Historical records from the Tonle Sap basin were noted based on specific mentions of ‘Tonle Sap Lake’ or ‘grand lac’, and localities within the provinces around the Lake, i.e., Kampong Chhnang, Pursat, Battambang, Banteay Meanchey, Siem Reap and Kampong Thom.

### Sampling

**Figure 1.**
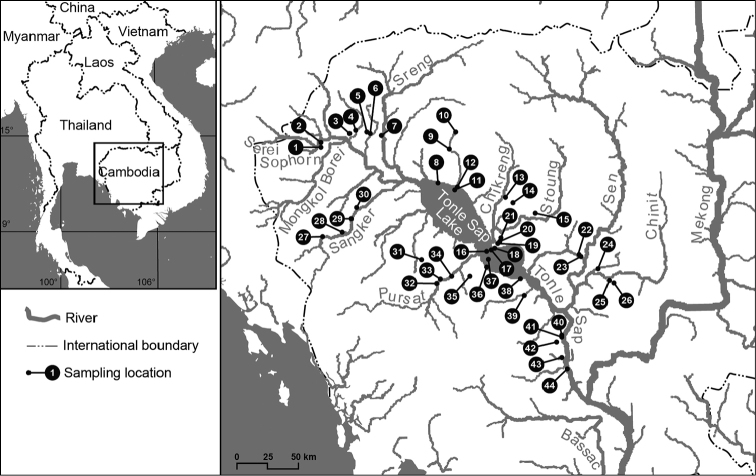
Map of sampling localities of freshwater molluscs within the Tonle Sap basin, Cambodia. The numbers indicate collection sites that correspond to Table 1. Inset shows location of the focal area within the Indochinese region.

We conducted surveys in and around the Tonle Sap Lake and its major tributaries over two one-week periods in May and December 2019, respectively. Samples were taken from 44 locations from the study area, covering major landing sites (Chhnouk Tru in Kampong Chhnang Province, Kampong Loung in Pursat Province, Chong Khneas in Siem Reap Province and Boeung Chhmar Ramsar wetlands in Kampong Thom Province), major Tonle Sap tributaries (Pursat, Sangkae, Mongkol Borei, Serei Saophoan, Sreng, Chi Kraeng, Staung, Sen, and Chinit Rivers), rain-fed and flooded zones along national road No. 5 and 6 around the Tonle Sap Lake (Fig. 1, Table 1). Specimens were collected by hand and with long-handled nets. Some specimens were also purchased from local markets.

**Table 1.** Sampling localities of freshwater molluscs collected from the Tonle Sap basin in 2019.

No.	Locality code and locality details	Coordinates
1	C078-Serei Saophoan River, Serei Saophoan city, Banteay Meanchey Province	13°35'08.9"N, 102°58'41.2"E
2	C073-Serei Saophoan River, Preah Ponlea, Serei Saophoan District, Banteay Meanchey Province	13°34'33.1"N, 102°58'58.5"E
3	C017-Paoy Samraong, Preah Netr Preah, Preah Netr Preah District, Banteay Meanchey Province	13°35'01.8"N, 103°09'24.8"E
4	C075-River in Chob Vari, Preah Netr Preah District, Banteay Meanchey Province	13°37'24.0"N, 103°11'50.0"E
5	C080-Lotus pond in Phnum Lieb, Preah Netr Preah District, Banteay Meanchey Province	13°36'31.8"N, 103°18'02.3"E
6	C079-River in Phnum Lieb, Preah Netr Preah District, Banteay Meanchey Province	13°36'16.9"N, 103°19'02.0"E
7	C074-Sreng River in Kralanh City, Kralanh District, Siem Reap Province	13°35'29.8"N, 103°24'15.8"E
8	C018-Tonle Sap Lake open area near Chong Khneas, Chong Khneas, Siem Reap Province	12°30'20.1"N, 103°50'06.2"E
9	C085-Siem Reap River, Leang Dai, Angkor Thom District, Siem Reap Province	13°29'24.3"N, 103°55'14.4"E
10	C081-Stream near Banteay Srei District, Siem Reap Province	13°35'44.0"N, 103°57'43.3"E
11	C020-Tonle Sap Lake open area near Kampong Phluk, Prasat Bakong District, Siem Reap Province	13°11'16.2"N, 103°57'25.4"E
12	C021-Tonle Sap Lake open area near Kampong Phluk, Prasat Bakong District, Siem Reap Province	13°11'40.6"N, 103°57'50.5"E
13	C082-Chi Kraeng River in Kampong Kdei, Chi Kraeng District, Siem Reap Province	13°07'55.3"N, 104°20'16.8"E
14	C023-Ponds in Thnol Keng, Kampong Kdei, Chi Kraeng District, Siem Reap Province	13°05'25.4"N, 104°23'32.5"E
15	C024-Paddy fields near Trach, Kampong Chen Cheung, Stoung District, Kampong Thom Province	12°58'43.7"N, 104°33'52.3"E
16	C009-Tonle Sap Lake open area in Kampong Thom Province	12°42'42.5"N, 104°11'58.2"E
17	C008-Tonle Sap Lake open area near Pean Bang, Stoung District, Kampong Thom Province	12°43'36.9"N, 104°14'11.6"E
18	C007-River flows from Boeng Tonle Chhma to Tonle Sap Lake, Stoung District, Kampong Thom Province	12°44'15.4"N, 104°15'30.9"E
19	C004-Don Sdeung, Peam Bang, Staung District, Kampong Thom Province	12°46'25.4"N, 104°17'02.9"E
20	C005-Don Sdeung, Boeung Chhmar Fish Sanctuary, Staung District, Kampong Thom Province	12°47'25.1"N, 104°17'55.7"E
21	C006-Provincial Fisheries Office, Boeung Chhmar Fish Sanctuary, Staung District, Kampong Thom Province	12°48'48.6"N, 104°18'11.6"E
22	C087-Sen River in Balang, Damrei Choan Khla, Stung Sen District, Kampong Thom Province	12°41'48.5"N, 104°53'58.8"E
23	C025-Sen River in Kampong Samraung, Srayav, Stung Sen District, Kampong Thom Province	12°40'58.3"N, 104°54'33.1"E
24	C090-Tang Krasang River in Santuk District, Kampong Thom Province	12°34'04.4"N, 105°03'02.3"E
25	C092-Chinit River in Kampong Thma, Santuk District, Kampong Thom Province	12°29'45.7"N, 105°07'18.4"E
26	C088-Canal from Makara Dam, Ballangk, Baray District, Kampong Thom Province	12°29'13.8"N, 105°08'47.0"E
27	C068-Recreational area, Sangkae River in Traeng, Rotanak Mondol, Battambang Province	12°49'51.4"N, 102°55'44.3"E
28	C071-Sangkae River in Chaeng Mean Chey, Banan District, Battambang Province	12°52'03.5"N, 103°06'03.8"E
29	C070-Sangkae River in Chheu Teal, Banan District, Battambang Province	12°58'54.7"N, 103°08'36.2"E
30	C014-Sangkae River in Wat Ta Meum, Oudambang Muoy, Sangkae District, Battambang Province	13°04'04.7"N, 103°12'17.2"E
31	C067-River in Ou Ta Paong, Bakan District, Pursat Province	12°39'34.8"N, 103°40'26.2"E
32	C013-Pursat River near Damnak Ampel Irrigation Dam, Damnak Ampel, Lolork Sor, Sampaov Meas District, Pursat Province	12°29'19.3"N, 103°48'29.7"E
33	C012-Pursat River in Wat Loung, Lolork Sor, Sampaov Meas District, Pursat Province	12°30'20.1"N, 103°50'07.8"E
34	C064-Pursat River in Sorya, Krong Pursat, Pursat Province	12°31'00.3"N, 103°54'54.7"E
35	C066-Thliem Ma-Orm River in Boeng Kantuot, Krakor District, Pursat Province	12°31'37.3"N, 104°03'13.8"E
36	C011-Tonle Sap Lake open area in Krakor District, Pursat Province	12°35'42.1"N, 104°12'13.1"E
37	C010-Tonle Sap Lake open area in Krakor District, Pursat Province	12°38'20.2"N, 104°12'12.1"E
38	C003-Kampong Chhnok Tru landing point, Chhnok Tru, Boribo District, Kampong Chhnang Province	12°30'36.5"N, 104°27'18.2"E
39	C063-Tributary of Tonle Sap Lake in Phumi Phsar, Kampong Chhnang Province	12°22'50.7"N, 104°29'0.2"E
40	C002-Tonle Sap River in Kampong Prasat, Saeb, Kampong Tralach District, Kampong Chhnang Province	12°04'23.6"N, 104°46'23.8"E
41	C097-Tonle Sap River in Kaoh Thkov, Chol Kiri District, Kampong Chhnang Province	12°03'31.7"N, 104°46'22.4"E
42	C001-Boeung Po, Sorvong, Tacheise, Kampong Tralach District, Kampong Chhnang Province	12°01'28.3"N, 104°43'54.1"E
43	C094-Tonle Sap River in Samretthi Chey, Kampong Tralach district, Kampong Chhnang Province	11°53'55.2"N, 104°46'01.6"E
44	C093-Tonle Sap River in Kaoh Chen, Popnhea Lueu district, Kandal Province	11°49'01.6"N, 104°48'40.5"E

### Species identification

All the specimens were identified to genus or species level based on shell characteristics by referring to the historical literature with original species descriptions, Brandt (1974), Morton and Dinesen (2010), Jeratthitikul et al. (2019a, b) and Muanta et al. (2019). The annotated species checklist is based on freshly collected specimens in this study only, and is organised according to higher classification (class, subclass or equivalent, order, superfamily), family, and species, in alphabetical order. Higher classifications follow Lydeard and Cummings (2019), and valid names mainly follow MolluscaBase (2019) and Graf and Cummings (2019).

Voucher specimens are deposited in the following institutions:

**CIFI** Inland Fisheries Research and Development Institute, Cambodia;

**MUMNH** Mahidol University Museum of Natural History, Thailand;

**ZRC**Zoological Reference Collection of the Lee Kong Chian Natural History Museum, National University of Singapore.

## Results

### Historical records for Cambodia

Almost 300 species of freshwater molluscs have previously been recorded from Cambodia to date, but only 153 are currently considered to be valid species and among these, 33 species were recorded from the Tonle Sap basin (Table 2, Suppl. material 1: Table S1), including records mentioning Battambang Province and Kampong Svay in Kampong Thom Province (as ‘Campong/Kompong-Soai’ in Suppl. material 1: Table S1). Two species are noted to have uncertain or doubtful presence in Cambodia. The first, *Scaphula pinna* Benson, 1856, is present in the southern Mekong delta in Vietnam, and is thus presumed to be extant in Cambodia as well (Madhyastha 2012; but see species account for *Scaphula minuta* in the next section). Secondly, *Paludina fulva* Benson, 1863, described from Cambodia, was synonymised with *Idiopoma dissimilis* (OF Müller, 1774) by Brandt (1974), who recognised the species as being distributed from India to northern Thailand, and hence regarded the record from Cambodia to be doubtful. Among the historical records with known taxonomic issues are three species that are not recognised as valid in MolluscaBase (2019), i.e., *Filopaludina danieli* (Morlet, 1889), *Filopaludina obscurata* (Deshayes & Jullien, 1876) and *Mekongia paviei* (Morlet, 1889), and one species, although with an accepted name, i.e., *Mekongia turbinata* (Deshayes & Jullien, 1876), has been highlighted as requiring taxonomic revision (Köhler and Rintelen 2011).

A third of the previously recorded species are medically-important Pomatiopsidae and Stenothyridae, which are intermediate hosts of zoonotic parasites like *Schistosoma mekongi* Voge, Bruckner & Bruce, 1978, and are distributed in the Mekong River (Davis 1979; Attwood et al. 2004). One hundred and six species have had their conservation status assessed by the IUCN (2018), and among them, 66 species are Least Concern, 32 species are Data Deficient, and eight species of Pomatiopsidae have been assessed as Vulnerable or Near-Threatened (Suppl. material 1: Table S1).

### Preliminary survey of Tonle Sap Lake and its watershed

At least 15 species of bivalves from five families, and 16 species of gastropods from eight families (Table 2) were collected from around Tonle Sap Lake and the surrounding habitats, including tributaries, paddy fields, and ponds.

**Table 2.** Summary of freshwater molluscs recorded in historical records of Cambodia, historical records that specify the Tonle Sap basin, and collected from the Tonle Sap basin in 2019. Higher classification follows Lydeard and Cummings (2019).

Higher classification	Order	Superfamily	Family	Number of species		
				Historical records of Cambodia	Historical records mentioning Tonle Sap basin	Present study of Tonle Sap basin
** Bivalvia **						
Pteriomorphia	Arcida	Arcoidea	Arcidae	1	–	1
	Mytilida	Mytiloidea	Mytilidae	2	2	2
Heterodonta	Adapedonta	Solenoidea	Pharidae	–	–	1
	Venerida	Cyrenoidea	Cyrenidae	14	6	> 1
Palaeoheterodonta	Unionida	Unioidea	Unionidae	40	13	10
** Gastropoda **						
Caenogastropoda	Architaenioglossa	Ampullarioidea	Ampullariidae	7	4	4
		Viviparoidea	Viviparidae	19	3	4
	Littorinimorpha	Truncatelloidea	Bithyniidae	4	1	2
			Iravadiidae	1	–	–
			Pomatiopsidae	46	–	–
			Stenothyridae	6	–	–
	Neogastropoda	Buccinoidea	Nassariidae	7	2	2
	(Cohort) Sorbeoconcha	Cerithioidea	Pachychilidae	2	1	1
			Thiaridae	2	1	1
Heterobranchia						
Hygrophila		Lymnaeoidea	Bulinidae	1	–	1
			Lymnaeidae	1	–	1
**Total**				153	33	31

### Annotated checklist of freshwater molluscs recorded from Tonle Sap basin

#### Class Bivalvia Linnaeus, 1758

##### Subclass Pteriomorphia Beurlen, 1944

###### Order Arcida Stoliczka, 1871

**Superfamily Arcoidea Lamarck, 1809**

**Family Arcidae Lamarck, 1809**

####### Scaphula minuta

Taxon classificationAnimaliaArcidaArcidae

Ghosh, 1922

18557EB9-1366-5A4F-8DB8-E7918552317D

Fig. 2A

Scaphula minuta Ghosh, 1922: 1143–1144. Type locality: “Tale Sap or inland Sea of Singgora on the east coast of peninsular Siam”.

######## Material examined.

CIFI.MOL.044, ZRC.MOL.015755.

######## Distribution and habitat.

Pursat River in Pursat Province, and Sreng River in Siem Reap Province (locality no. 7 and 32). Found attached to rocks in slow-moving waters.

######## Remarks.

*Scaphula minuta* was first described from Songkhla Lake in southern Thailand (Ghosh 1929) and only recently reported to be distributed in southern Vietnam (Bogan and Do 2014). In addition, Bogan and Do (2014) mentioned that past records of ‘*Scaphula pinna*’ in Thailand (i.e., Brandt 1974) should be referred to as *Scaphula minuta*. Based on Bogan and Do (2014), the species previously identified as *Scaphula pinna* and cited as being uncertain in Cambodia (Madhyastha 2012) was probably *Scaphula minuta*. However, the distinction between these two species is pending systematic revision, and our records represent the first confirmation of *Scaphula minuta* in Cambodia.

**Figure 2.**
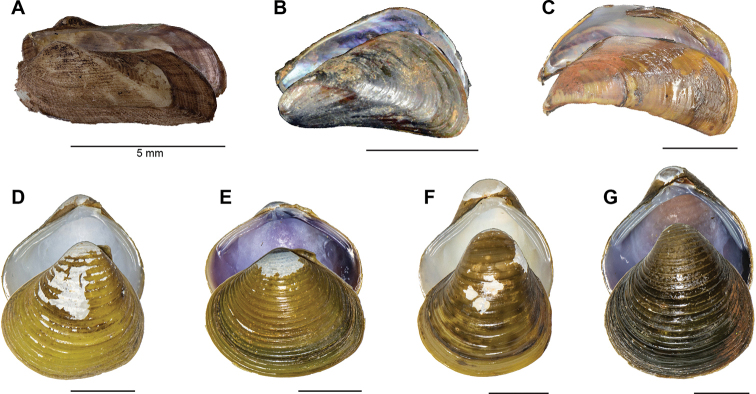
Freshwater bivalves of the Tonle Sap basin, Cambodia (Arcidae, Mytilidae, Cyrenidae) **A***Scaphula minuta***B***Limnoperna fortunei***C***Sinomytilus harmandi***D–G***Corbicula* spp. Scale bars: 10 mm, unless stated otherwise. Photographs by TH Ng (**A–C**) and K Macharoenboon (**D–G**).

###### Order Mytilida Férussac, 1822

**Superfamily Mytiloidea Rafinesque, 1815**

**Family Mytilidae Rafinesque, 1815**

####### Limnoperna fortunei

Taxon classificationAnimaliaMytilidaMytilidae

(Dunker, 1856)

EE6ACAC1-C051-5E6D-B980-FD3C4EE01C47

Fig. 2B

Volsella fortunei Dunker, 1856: 361, 362. Type locality: “Mare Chinense”.
Limnoperna siamensis : Brandt 1974: 256.
Limnoperna fortunei : Morton and Dinesen 2010: 57–72.

######## Material examined.

ZRC.MOL.015660, ZRC.MOL.015661, ZRC.MOL.015664, ZRC.MOL.015665, ZRC.MOL.015666.

######## Distribution and habitat.

Tonle Sap River at Kampong Chhnang Province, and Tonle Sap Lake at Kampong Chhnang and Siem Reap Provinces (locality no. 8, 12, 37, 38 and 40). Occur in colonies attached by byssus threads to hard surfaces like man-made structures (e.g., boats, jetties, wooden pillars of homes), on unionid bivalves, and occasionally the shells of large freshwater gastropods.

######## Remarks.

*Limnoperna fortunei* is native to East Asia, and although previously thought to be naturally distributed in the countries south of China (e.g., Brandt 1974), has recently been proposed to be introduced to tropical Indochina, including Cambodia (Morton and Dinesen 2010). However, its occurrence in Cambodia has been noted since the latter half of the 1800s (Suppl. material 1: Table S1, also see Morton and Dinesen (2010)). The species is known to be sympatric with *Sinomytilus harmandi* in Cambodia (Morton and Dinesen 2010), and in fact, we found that both species are syntopic, as most colonies of *Limnoperna fortunei* included individuals of *Sinomytilus harmandi*.

####### Sinomytilus harmandi

Taxon classificationAnimaliaMytilidaMytilidae

(Rochebrune, 1882)

82A5D783-374E-5197-B991-BC419286B01E

Fig. 2C

Dreissena harmandi Rochebrune, 1882: 102. Type locality: “Lac de Rhom-Penh, Mekong”.
Sinomytilus harmandi : Brandt 1974: 307, pl. 26, fig. 69; Morton and Dinesen 2010: 57–72.

######## Material examined.

ZRC.MOL.015657, ZRC.MOL.015658, ZRC.MOL.015659, ZRC.MOL.015667, ZRC.MOL.015668.

######## Distribution and habitat.

Similar to that of *Limnoperna fortunei*.

######## Remarks.

*Sinomytilus harmandi* appears to be limited in range to the Lower Mekong River basin, and it may have previously been mistaken for *Limnoperna fortunei*, resulting in a lack of historical records (Morton and Dinesen 2010). Our finding that *Sinomytilus harmandi* often co-occurs with *Limnoperna fortunei*, but in much lower densities, may further account for the former being overlooked in the literature. *Sinomytilus harmandi* may be distinguished from *Limnoperna fortunei* by the presence of an interior shell septum.

##### Subclass Heterodonta Meumayr, 1884

###### Order Adapedonta Cossmann & Peyrot, 1909

**Superfamily Solenoidea Lamarck, 1809**

**Family Pharidae H. Adams & A. Adams, 1856**

####### Novaculina siamensis

Taxon classificationAnimaliaAdapedontaPharidae

Morlet, 1889

5B1BFE0D-54F1-5DFD-88B4-D3D2C5B724BC

Fig. 3A

Novaculina siamensis Morlet, 1889: 198, pl. 9, fig. 4. Type locality: “Marais de Chantakam Siam”.

######## Material examined.

MUMNH.PHA.001, MUMNH.PHA.002

######## Distribution and habitat.

Tonle Sap River in Kampong Chhnang Province (locality no. 41 and 43); tough clay bottom substrate, in which it makes cylindrical holes.

######## Remarks.

The discovery of *Novaculina siamensis* in Tonle Sap River is a new record for Cambodia and fills in the distribution gap of the genus in Indochina. This species was first described from “Chantakam, Siam” [Prachantakham District, Prachinburi Province, Thailand], but the type series is thought to be lost (Bolotov et al. 2018). The type locality is probably a tributary of Bang Pakong River, in eastern Thailand. Brandt (1974) reported an additional population from Pasak River in Thailand. Sayenko et al. (2017) reported another abundant population from the Mekong Delta in Vietnam. The Tonle Sap River population is smaller, with more prominent umbo and lower posteriorly than the Thai population. All the collected shells were found empty, inside their cylindrical holes, under the shallow water near the riverbank. Only one living animal was obtained.

**Figure 3.**
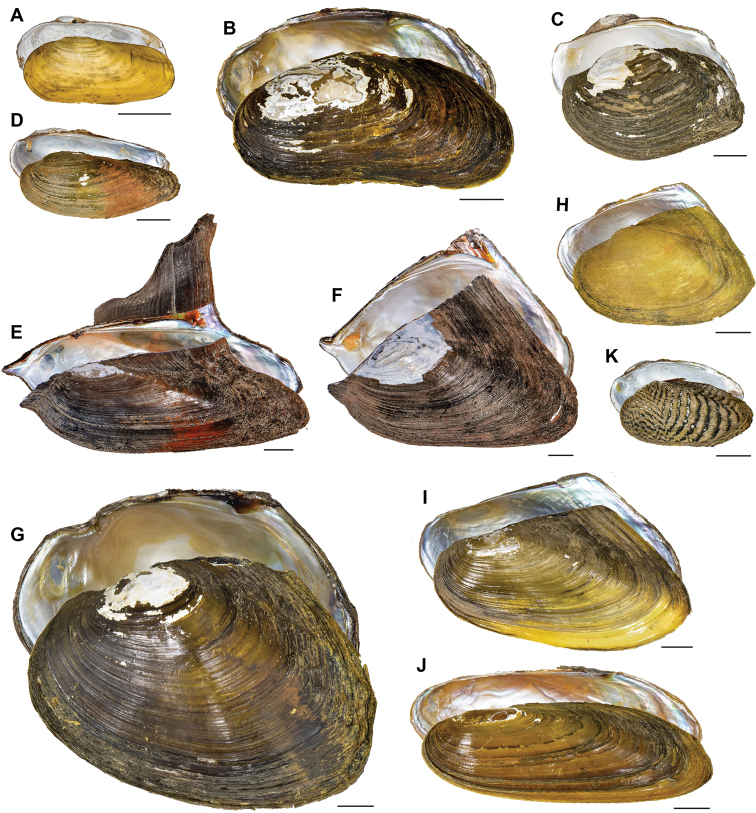
Freshwater bivalves of the Tonle Sap basin, Cambodia (Pharidae and Unionidae) **A***Novaculina siamensis***B***Bineurus mouhotii***C***Contradens contradens***D***Ensidens ingallsianus***E***Hyriopsis bialata***F***Hyriopsis delaportei***G***Monodontina cambodjensis***H***Physunio micropterus***I***Pilsbryoconcha linguaeformis***J***Pilsbryoconcha lemeslei* and **K***Scabies mandarinus*. Scale bars: 10 mm, unless stated otherwise. Photographs by K Macharoenboon.

###### Order Venerida Gray, 1854

**Superfamily Cyrenoidea Gray, 1840**

**Family Cyrenidae Gray, 1840**

####### Corbicula spp.

Taxon classificationAnimaliaVeneridaCyrenidae

8F82BB7B-A30B-50A7-922C-D6A6827A1AD1

Fig. 2D–G

######## Material examined.

CIFI.MOL.029, CIFI.MOL.030, CIFI.MOL.031, CIFI.MOL.032, MUMNH.COR.0194, MUMNH.COR.0195, MUMNH.COR.0196, MUMNH.COR.0197, MUMNH.COR.0198, MUMNH.COR.0199, MUMNH.COR.0200, MUMNH.COR.0201, MUMNH.COR.0202, MUMNH.COR.0203, MUMNH.COR.0204, MUMNH.COR.0205, MUMNH.COR.0206, MUMNH.COR.0207, MUMNH.COR.0208, MUMNH.COR.0209, MUMNH.COR.0210, MUMNH.COR.0211, MUMNH.COR.0212, ZRC.MOL.015634, ZRC.MOL.015635, ZRC.MOL.015636, ZRC.MOL.015637, ZRC.MOL.015638.

######## Distribution and habitat.

Tonle Sap River and Lake, and most tributaries (locality no. 3, 6, 7, 8, 11, 12, 16, 18, 19, 20, 21, 22, 23, 24, 27, 32, 38, 39 and 40). Buried just below surface of soft, muddy substrate of the water bodies.

######## Remarks.

Approximately 17 different species of *Corbicula* have previously been recorded from Cambodia (Suppl. material 1: Table S1), but there has been no known attempt to verify if all are valid records. In neighbouring Thailand, 21 nominal *Corbicula* species were all found to belong to only *Corbicula fluminea* (OF Müller, 1774), a single species (Kijviriya et al. 1991). Based on morphological examination of the fresh material from the Tonle Sap basin, it is possible that there may be more than one *Corbicula* species present. However, further investigation including molecular analysis will be required to confirm the species identities (Bieler and Mikkelsen 2019).

**Figure 4.**
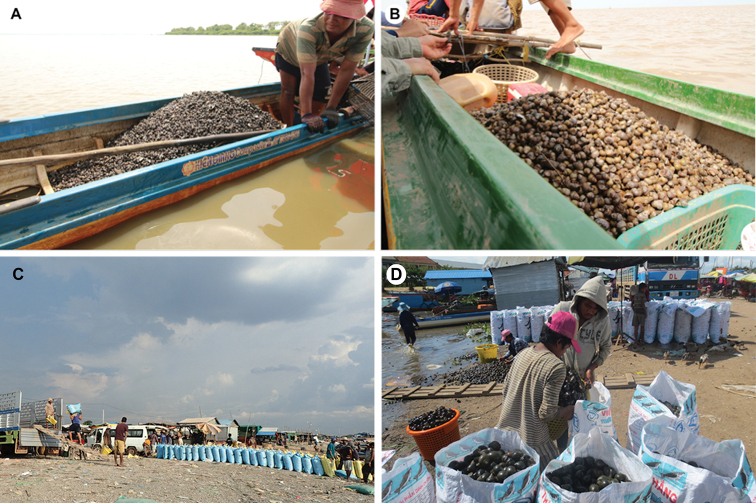
Freshwater mollusc fisheries at the Tonle Sap Lake, Cambodia. Small-scale harvesting of freshwater molluscs **A***Corbicula* spp. and **B***Mekongia rattei*, at open areas of the Lake. Freshwater molluscs **C***Corbicula* spp. and **D***Pila* spp., being sorted and packed at main landing sites around the Lake. Photographs by A Pholyotha (**A, B**), TH Ng (**C**), PB Ngor (**D**).

*Corbicula* species are the only bivalves that are commercially harvested from the Lake to be sold locally and exported abroad for human consumption and as animal feed (Fig. 4A, C). More than 6000 tonnes of *Corbicula* clams were recorded from five commercial landing sites in Kampong Chhnang Province, within the Tonle Sap basin, over the period of one year (Ngor et al. 2018d).

##### Subclass Palaeoheterodonta Newell, 1965

###### Order Unionida Gray, 1854

**Superfamily Unionoidea Rafinesque, 1820**

**Family Unionidae Rafinesque, 1820**

####### Bineurus mouhotii

Taxon classificationAnimaliaUnionidaUnionidae

(Lea, 1863)

44B753DC-50D2-53DC-985C-A963B874A69A

Fig. 3B

Monocondylaea mouhotii Lea, 1863: 190. Type locality: “Laos Mountains, Cambodia, Siam”.
Pseudodon mouhoti : Brandt 1974: 265–266, pl. 19, fig. 25
Bineurus mouhotii : Pfeiffer et al. 2019: 116.

######## Material examined.

MUMNH.UNI.2637, MUMNH.UNI.2646, MUMNH.UNI.2659 MUMNH.UNI.2670.

######## Distribution and habitat.

Phumi Phsar River, Kampong Chnnang Province and Sangkae River, Battambang Province (locality no. 27 and 39); in sandy substrate of streams.

######## Remarks.

*Bineurus mouhotii* is widespread along the Mekong basin in Thailand, Laos and Vietnam, and probably in northern Myanmar and Yunnan (Brandt 1974). The shell shape of present specimens was very inequilateral with concave ventral margin, which is a common form of the mountain race. The ventral margin of specimens collected from the downstream section is usually less concave, and nearly straight or even slightly curved (Brandt 1974). The combination of *Bineurus mouhotii* is a recent revision, and first appeared in Bolotov et al. (2017). The validity of the genus *Bineurus* was latter confirmed by phylogenomic analysis (Pfeiffer et al. 2019). However, the complete systematic revision of this taxon (and other related species previously included in the genus *Pseudodon* sensu Brandt, 1974) has yet to be investigated.

####### Contradens contradens

Taxon classificationAnimaliaUnionidaUnionidae

(Lea, 1838)

C5815A72-C5AB-5BD7-9EE7-26ABDB04D630

Fig. 3C

Unio contradens Lea, 1838: 75, pl. 18, fig. 58. Type locality: Java.
Uniandra contradens tumidula : Brandt 1974: 290–291, pl. 24, figs 51, 52.
Uniandra contradens rustica : Brandt 1974: 291–292, pl. 24, fig. 53.
Uniandra contradens fischeriana : Brandt 1974: 292, pl. 24, fig. 55.

######## Material examined.

CIFI.MOL.017, CIFI.MOL.018, MUMNH.UNI.2621, MUMNH.UNI.2629, MUMNH.UNI.2633, MUMNH.UNI.2648, MUMNH.UNI.2651, ZRC.MOL.015639.

######## Distribution and habitat.

Tonle Sap Lake at Kampong Chhnang and Siem Reap Provinces, and Chhnok Tru landing point (locality no. 8, 11, 18, 36, 37, and 39); in soft muddy substrate.

######## Remarks.

Not sold for food, collected as by-catch of *Corbicula* and *Mekongia* harvests, and were often observed to be discarded along with other large unionids. *Contradens contradens* was recently noted for its high varation in shell morphology dued to phenotypic plasticity (Jeratthitikul et al. 2019b). The species recognised here may represent one of *Contradens contradens* varations or could be recognised as a distinct species, e.g., *Unio dautzenbergi* Morlet, 1889. Further molecular studies are necessary to confirm the taxonomic status. The present collected specimens are strongly sculptured with irregularly concentric wrinkles throughout the shell, similar to some populations from Chao Praya basin, Thailand.

####### Ensidens ingallsianus

Taxon classificationAnimaliaUnionidaUnionidae

(Lea, 1852)

31C70802-B231-5FC0-A624-8DEA20589506

Fig. 3D

Unio ingallsianus Lea, 1852: 282, pl. 24, fig. 41. Type locality: “Siam”.
Ensidens ingallsianus ingallsianus : Brandt 1974: 288, pl. 24, fig. 47.
Ensidens ingallsianus : Muanta et al. 2019: 224–231.

######## Material examined.

CIFI.MOL.019, CIFI.MOL.020, CIFI.MOL.021, CIFI.MOL.022, MUMNH.UNI.2617, MUMNH.UNI.2626, MUMNH.UNI.2634, MUMNH.UNI.2642, MUMNH.UNI.2644, MUMNH.UNI.2649, MUMNH.UNI.2655, MUMNH.UNI.2657, MUMNH.UNI.2666, MUMNH.UNI.2668, MUMNH.UNI.2672, MUMNH.UNI.2673, ZRC.MOL.015640, ZRC.MOL.015641, ZRC.MOL.015642, ZRC.MOL.015643.

######## Distribution and habitat.

Tonle Sap Lake at Kampong Chhnang, Siem Reap and Kampong Thom Provinces, Sen River in Kampong Thom Province, Sangkae River in Battambang Province, Chi Kraeng River and Kralanh River in Siem Reap Province, Tonle Sap River in Kandal Province (locality no. 3, 7, 8, 12, 13, 21, 23, 24, 30, 36, 40 and 44); in soft muddy substrate.

######## Remarks.

Not sold for food, collected as by-catch of *Corbicula* and *Mekongia* harvests, and were often observed to be discarded along with other large unionids. The species previously identified as *Ensidens ingallsianus* has recently been revealed as a complex of two species (Muanta et al. 2019), one clade that is restricted to the Chao Praya River basin in Thailand, and another clade from the Tonle Sap and Bang Prakong River basins (Muanta et al. 2019). These two clades could be separated by using the position of the umbo, which is more anterior than in the Tonle Sap clade. As far as we know, there is no other available name for the species from the Tonle Sap basin. Therefore, the name *Ensidens ingallsianus* is herein used until the systematic revision of this species complex is completed.

####### Hyriopsis bialata

Taxon classificationAnimaliaUnionidaUnionidae

(Simpson, 1900)

CA617F2C-6AB1-50F8-8E80-ECF991556472

Fig. 3E

Unio delphinus Gruner, 1841: 276, pl. 9, fig. 1a–c. (non Spengler, 1793) Type locality: “sungi flumine, Malaccae”.
Hyriopsis bialatus Simpson, 1900: 579. (new replacement name for Unio delphinus).
Hyriopsis (Hyriopsis) bialatus : Brandt 1974: 272–273, pl.21, fig. 36.

######## Material examined.

CIFI.MOL.007, CIFI.MOL.008, CIFI.MOL.009, MUMNH.UNI.2618, MUMNH.UNI.2622, MUMNH.UNI.2627, MUMNH.UNI.2630, MUMNH.UNI.2635, MUMNH.UNI.2650, MUMNH.UNI.2652, MUMNH.UNI.2658, MUMNH.UNI.2662, ZRC.MOL.015644.

######## Distribution and habitat.

Tonle Sap River in Kampong Chhnang Province and Tonle Sap Lake at Kampong Chhnang and Siem Reap Provinces, Sen River in Kampong Thom Province, Sangkae River, Battambang Province (locality no. 8, 11, 16, 23, 27, 36, 37, 38 and 40); in soft muddy substrate.

######## Remarks.

Not sold for food, collected as by-catch of *Corbicula* and *Mekongia* harvests, and were often observed to be discarded along with other large unionids. At some parts of the Tonle Sap Lake, shells of *Hyriopsis bialata* were often covered in mats of *Limnoperna fortunei* and *Sinomytilus harmandi*. *Hyriopsis bialata* is widespread in Indochina, from Thailand to Peninsular Malaysia, and along the middle Mekong basin to the Mekong Delta in southern Vietnam (Brandt 1974). Recent molecular analyses have revealed cryptic divergence in *Hyriopsis bialata* based on specimens from peninsular Malaysia and the Chao Phraya and Mekong basins (Zieritz et al. 2016). Pending systematic revision of this species complex, the name *Hyriopsis bialata* is herein used for the species in Cambodia.

####### Hyriopsis delaportei

Taxon classificationAnimaliaUnionidaUnionidae

(Crosse & Fischer, 1876)

F3762FD6-894B-59FB-8509-057E7A4CA748

Fig. 3F

Unio (Arconaia) delaportei Crosse & Fischer, 1876: 327–329, pl. 10, fig. 1, pl. 11, fig. 5. Type locality: “Cambodge, dans la province de Compong-Soai”.
Hyriopsis (Hyriopsis) delaportei : Brandt 1974: 273–274, pl. 21, fig. 37.

######## Material examined.

CIFI.MOL.025, CIFI.MOL.026, MUMNH.UNI.2623, MUMNH.UNI.2628, MUMNH.UNI.2631, MUMNH.UNI.2653, ZRC.MOL.015645.

######## Distribution and habitat.

Tonle Sap Lake at Kampong Chhnang, Kampong Thom, Pursat and Siem Reap Provinces (locality no. 11, 16, 37 and 38); in soft muddy substrate.

######## Remarks.

Not sold for food, collected as by-catch of *Corbicula* and *Mekongia* harvests, and were often observed to be discarded along with other large unionids. Shells of *Hyriopsis delaportei* were also found to be covered in mats of *Limnoperna fortunei* and *Sinomytilus harmandi*. *Hyriopsis delaportei* was originally described from “Compong Soai; Cambodia” (Morlet 1889). It is abundant in the Tonle Sap Lake and its tributaries. The distribution range outside the country is in Vietnam in the Mekong River delta at An Giang, close to the border with Cambodia (Bogan and Do 2014), and in Thailand at the Satung River, Srakaeo and Kaek River, Pitsanulok, although the latter record remains uncertain (Brandt 1974).

####### Monodontina cambodjensis

Taxon classificationAnimaliaUnionidaUnionidae

(Petit de la Saussaye, 1865)

E8DE255F-0B08-57C0-908F-29CC6E7130CA

Fig. 3G

Monocondylaea cambodjensis Petit de la Saussaye, 1865: 16, pl. 4, fig. 4. Type locality: “Battabbang, Cambodge”.
Pseudodon cambodjensis cambodjensis : Brandt 1974: 269, pl. 19, fig. 28.
Monodontina cambodjensis : Bolotov et al. 2017: 11573.

######## Material examined.

CIFI.MOL.023, CIFI.MOL.024, MUMNH.UNI.2637, MUMNH.UNI.2646, MUMNH.UNI.2659, MUMNH.UNI.2670, ZRC.MOL.015652, ZRC.MOL.015653, ZRC.MOL.015654.

######## Distribution and habitat.

Tonle Sap River in Kampong Chhnang Province, Pursat River in Pursat Province and Sen River in Kampong Thom Province, Chi Kraeng River in Siem Reap Province (locality no. 3, 13, 23 and 33); in soft muddy or sandy substrate.

######## Remarks.

Not sold for food, collected as by-catch of *Corbicula* and *Mekongia* harvests, and were often observed to be discarded along with other large unionids. *Monodontina cambodjensis* has been recorded from Thailand and Cambodia, in several tributaries of the Mekong River (Brandt 1974). Bolotov et al. (2017) resurrected the genus *Monodontina* Conrad, 1853 and used it as a generic name for *Monodontina vondembuschiana* (Lea, 1840) and *Monodontina cambodjensis*. This taxonomic opinion was based solely on molecular evidence, without any morphological revision. *Monodontina cambodjensis* is distinguishable from its similar congener, *Monodontina vondembuschiana*, by its high posterior wing and the rounded triangular shape.

####### Physunio micropterus

Taxon classificationAnimaliaUnionidaUnionidae

(Morelet, 1866)

8BFDF61A-4D0F-59AA-9232-A34AA40684ED

Fig. 3H

Unio micropterus Morelet, 1866: 63, 64. Type locality: “in torrentibus montanis Cambodiae”.
Physunio micropterus : Brandt 1974: 296–297, pl. 25, fig. 60.

######## Material examined.

CIFI.MOL.013, CIFI.MOL.014, CIFI.MOL.015, MUMNH.UNI.2639, MUMNH.UNI.2641, MUMNH.UNI.2656, MUMNH.UNI.2661, MUMNH.UNI.2665, MUMNH.UNI.2667, MUMNH.UNI.2671, ZRC.MOL.015646, ZRC.MOL.015647, ZRC.MOL.015648.

######## Distribution and habitat.

Pursat River in Pursat Province, Sangkae River in Battambang Province, Sen River in Kampong Thom Province, Chi Kraeng River and Sreng River in Siem Reap Province (locality no. 7, 13, 22, 23, 27, 30, 32 and 39); in sandy substrate.

######## Remarks.

The distribution of *Physunio micropterus* is restricted to the Tonle Sap basin. There are some reports outside its endemic range, such as in the Ping and Prachinburi rivers in Thailand, but these distributions need to be confirmed (Brandt 1974). Some specimens have been collected from Sai Khao river in Chanthaburi, Thailand (E Jeratthittikul, unpublished data), which flows into Cambodia, and finally drains into the Tonle Sap Lake.

####### Pilsbryoconcha linguaeformis

Taxon classificationAnimaliaUnionidaUnionidae

(Morelet, 1875)

90618220-15EE-545F-8479-ED17668BC798

Fig. 3I

Anodonta linguaeformis Morelet, 1875: 329, pl. 14, fig. 5. Type locality: “au Cambodje, probablement dans les marécages voisins de Battambang”.
Pilsbryoconcha linguaeformis : Simpson 1900: 587.
Pilsbryoconcha exilis linguaeformis : Brandt 1974: 265.

######## Material examined.

MUMNH.UNI.2616, MUMNH.UNI.2619, MUMNH.UNI.2624, MUMNH.UNI.2625, MUMNH.UNI.2636, MUMNH.UNI.2645 ZRC.MOL.015649, ZRC.MOL.015650, ZRC.MOL.015651.

######## Distribution and habitat.

Pond in Kampong Chhnang Province, Tonle Sap Lake in Kampong Chhnang and Kampong Thom Provinces, Banteay Meanchey Province, Sen River in Kampong Thom Province (locality no. 3, 20, 36, 38, 40, and 42); in soft muddy substrate and swampy grounds.

######## Remarks.

Not sold for food, collected as by-catch of *Corbicula* and *Mekongia* harvests, and were often observed to be discarded along with other large unionids. Distribution range of this species seems to be limited to the Tonle Sap basin. Brandt (1974) treated “*linguaeformis*” as a subspecies of *Pilsbryoconcha exilis* (Lea, 1838). Some authors included “*linguaeformis*” as a junior synonym of *Pilsbryoconcha carinifera* (Conrad, 1837) (e.g., Haas 1969). However, it differs from *Pilsbryoconcha exilis* and *Pilsbryoconcha carinifera* by the greater height of the posterior end.

####### Pilsbryoconcha lemeslei

Taxon classificationAnimaliaUnionidaUnionidae

(Morelet, 1875)

AA0C6EFC-070E-59D3-ABE8-499D987793F2

Fig. 3J

Anodonta lemeslei Morelet, 1875: 328, pl. 14, fig. 1. Type locality: “Cambodge”.
Pilsbryoconcha lemeslei : Brandt 1974: 263, pl. 18, fig. 22.

######## Material examined.

MUMNH.UNI.2669

######## Distribution and habitat.

Tributary of Tonle Sap Lake near Preah Tis Bridge in Chi Kraeng District, Siem Reap Province (locality no. 13); in soft muddy substrate of still water.

######## Remarks.

This species is rare and known only from few places in Thailand, Laos, Cambodia and Vietnam (Brandt 1974; Do et al. 2018). It differs from other *Pilsbryoconcha* species by having a narrower and elongated shell, and more rounded posterior end.

####### Scabies mandarinus

Taxon classificationAnimaliaUnionidaUnionidae

(Morelet, 1864)

BF891B10-8FF4-5AB8-A3C8-BC316548DDF4

Fig. 3K

Unio mandarinus Morelet, 1864: 159. Type locality: “Cochinchina”.
Scabies crispata : Brandt 1974: 281–282, pl. 20, fig. 33.
Scabies mandarinus : Pfeiffer et al. 2018: 403–413.

######## Material examined.

CIFI.MOL.001, CIFI.MOL.002, CIFI.MOL.003, CIFI.MOL.004, CIFI.MOL.004, CIFI.MOL.005, CIFI.MOL.006, MUMNH.UNI.2620, MUMNH.UNI.2632, MUMNH.UNI.2638, MUMNH.UNI.2640, MUMNH.UNI.2643, MUMNH.UNI.2647, MUMNH.UNI.2654, MUMNH.UNI.2663, MUMNH.UNI.2664, MUMNH.UNI.2672, ZRC.MOL.015655, ZRC.MOL.015656.

######## Distribution and habitat.

Tonle Sap Lake open area in Banteay Meanchey and Siem Reap Provinces, Tonle Sap River in Siem Reap Province and Chhnok Tru landing point, Sangkae River in Battambang Province, Pursat River in Pursat Province, Tang Krasang River in Kampong Thom Province, Sreng River in Siem Reap Province (locality no. 2, 3, 7, 8, 12, 24, 27, 30, 32, 36 and 38); in soft muddy and sandy substrate.

######## Remarks.

*Scabies mandarinus* was described by Morelet in 1864, based on specimens collected from “Cochinchina”. This name was previously placed as a junior synonym under *Scabies scobinatus* (Lea, 1856) and *Scabies crispata* (Gould, 1843) (e.g., Haas 1969; Brandt 1974). Based on molecular phylogeny data, Pfeiffer et al. (2018) revealed a distinct clade of Parreysiinae from Mekong delta and eastern gulf of Thailand and resurrected the name *Scabies mandarinus* for this clade. Jeratthitikul et al. (2019a) later included *Scabies* populations from the Tonle Sap basin into this species, based on molecular data and shell morphology.

#### Class Gastropoda Cuvier, 1795

##### Subclass Caenogastropoda Cox, 1960

###### Order Architaenioglossa Haller, 1890

**Superfamily Ampullarioidea Gray, 1824**

**Family Ampullariidae Gray, 1824**

####### Pila gracilis

Taxon classificationAnimaliaArchitaenioglossaAmpullariidae

(Lea, 1856)

E6A6B14D-99C9-55DB-9FC7-9724DD66F555

Fig. 5A

Ampullaria gracilis Lea, 1856: 110. Type locality: “Siam”.
Pila gracilis : Brandt 1974: 51–52, pl. 6, fig. 84.

######## Material examined.

ZRC.MOL.015669, ZRC.MOL.015670, ZRC.MOL.015690, ZRC.MOL.015673, ZRC.MOL.015677.

######## Distribution and habitat.

Tonle Sap River, Tonle Sap Lake open areas, and small ponds and paddy fields in the provinces surrounding the Lake (locality no. 4, 7, 9, 10, 24 and 28).

######## Remarks.

The first record of this species from Cambodia was from the Kampong Svay District in Kampong Thom Province (Crosse and Fischer 1876), which is slightly to the north of the Tonle Sap basin. This species is widely distributed in southern Thailand to central Malay peninsula, very rare in eastern Thailand, and also known from southern Vietnam (Brandt 1974; Ng et al. in press). *Pila gracilis* seemed to be rare in the eastern Thai provinces that border Cambodia (Ng et al. in press) but appears to be common in the Tonle Sap basin. Although *Pila gracilis* was not previously recorded as being harvested from the Tonle Sap Lake (Ngor et al. 2016, 2018d), we recorded *Pila gracilis* being sold together with *Pila pesmei* (Morlet, 1889) and *Pila virescens* (Deshayes, 1824) at one landing site in Kampong Chhnang Province, and collected *Pila gracilis* and *Pila virescens* from within the Lake.

**Figure 5.**
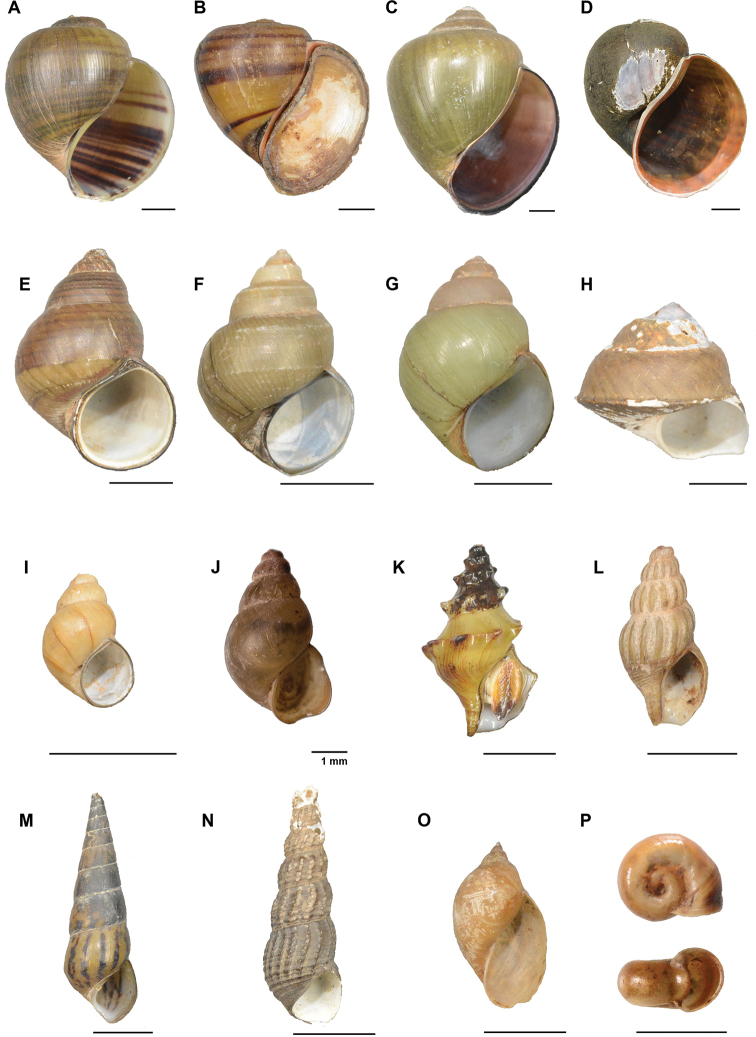
Freshwater gastropods of the Tonle Sap basin, Cambodia **A***Pila gracilis***B***Pila pesmei***C***Pila virescens***D***Pomacea maculata***E***Filopaludina martensi cambodjensis***F***Idiopoma umbilicata***G***Mekongia rattei***H***Trochotaia trochoides***I***Bithynia siamensis goniomphalus***J***Wattebledia siamensis***K***Anentome cambojiensis***L***Anentome helena***M***Sulcospira housei***N***Melanoides tuberculata***O***Indoplanorbis exustus* and **P***Radix rubiginosa*. Scale bars: 10 mm, unless stated otherwise. Photographs by TH Ng.

####### Pila pesmei

Taxon classificationAnimaliaArchitaenioglossaAmpullariidae

(Morlet, 1889)

0763C0E4-6C56-5B34-B1B1-64E7325ABBA1

Fig. 5B

Ampullaria pesmei Morlet, 1899: 185, pl. 8, fig. 2. Type locality: “Phnom-Penh (Cambodge)”.
Pila pesmei : Brandt 1974: 51, pl. 5, figs 70, 71.

######## Material examined.

ZRC.MOL.015671.

######## Distribution and habitat.

Tonle Sap open area (locality no. 38).

######## Remarks.

Although *Pila pesmei* is widely distributed in Eastern and Northeastern Thailand (Ng et al. in press), the species does not appear to be as widespread as *Pila gracilis* in the Tonle Sap basin, especially during the dry season when our surveys were conducted. It is also uncertain if the species is still extant at its type locality of Phnom Penh. *Pila pesmei* is harvested in abundance from the Tonle Sap Lake in the rainy season, with more than 380 tonnes recorded at five commercial landing sites in Kampong Chhnang Province within one year (as *Pila ampullacea* (Linneaus, 1758) in Ngor et al. 2016, 2018d). *Pila pesmei* may be confused with *Pila gracilis* owing to morphological similarity among some populations (Ng et al. in press), and further extensive examination of both species in Cambodia would be necessary to resolve the true distribution of both species in the country. The *Pila pesmei* morph from Tonle Sap Lake have relatively flat spires, compared to those in Thailand (Ng et al. in press), and appear to also be found in Southern Vietnam (as *Pila erythrochila* (Dautzenberg & Fischer, 1905) in Ng et al. 2019).

####### Pila virescens

Taxon classificationAnimaliaArchitaenioglossaAmpullariidae

(Deshayes, 1824)

E0B839BE-6282-5076-82F4-EE05D5098CEA

Fig. 5C

Ampullaria virescens Deshayes, 1824: un-numbered plate. Type locality: unknown.
Pila polita : Brandt 1974: 49, pl. 3, fig. 57.

######## Material examined.

CIFI.MOL.010, ZRC.MOL.015681, ZRC.MOL.015682, ZRC.MOL.015683, ZRC.MOL.015684, ZRC.MOL.015685, ZRC.MOL.015686, ZRC.MOL.015687, ZRC.MOL.015688, ZRC.MOL.015689.

######## Distribution and habitat.

Tonle Sap River and Lake, paddy fields and irrigation ponds in Kampong Chhnang, Banteay Meanchey and Siem Reap Provinces (locality no. 3, 13, 14, 18, 35, 38 and 42).

######## Remarks.

*Pila virescens* is the largest among the native ampullariids, and commonly harvested for food (Fig. 4D), and is often sold in markets and roadside stalls. An annual harvest of more than 1500 tonnes of the species was recorded from five commercial landing points in Kampong Chhnang Province (Ngor et al. 2018d).

####### Pomacea maculata

Taxon classificationAnimaliaArchitaenioglossaAmpullariidae

Perry, 1810

B6524F23-583F-51DC-B7FA-E87E07A28BB3

Fig. 5D

Pomacea maculata Perry, 1810: unnumbered plate and text. Type locality: Paraná, Argentina (see discussion in Hayes et al. 2012).

######## Material examined.

CIFI.MOL.037, ZRC.MOL.015691, ZRC.MOL.015695, ZRC.MOL.015692, ZRC.MOL.015693, ZRC.MOL.015694.

######## Distribution and habitat.

Tonle Sap River and Lake, and paddy fields in Banteay Meanchey and Kampong Thom Provinces (locality no. 1, 2, 5, 7, 15, 22, 38, 40, and 44).

######## Remarks.

Unlike *Pila* species, *Pomacea maculata* is not native to Southeast Asia. *Pomacea maculata* (as *Pomacea insularum* d’Orbigny, 1835 in Hayes 2008), and another species, *Pomacea canaliculata* (Lamarck, 1822), have both been introduced to Asia from South America (Hayes et al. 2012; Joshi et al. 2017). Molecular methods are the most accurate way to distinguish between them (Rama Rao et al. 2018), and DNA barcodes of two individuals from the Tonle Sap basin were a match to *Pomacea maculata* (GenBank Accession No. MT372328, MT372329). Because of the morphological similarity between the two species, some records of *Pomacea canaliculata* in Southeast Asia, including in Cambodia, may instead have been of *Pomacea maculata* (see Cowie and Hayes 2012). *Pomacea* species were first recorded in Cambodia only in the mid-1990s (compared to early 1980s in neighbouring Thailand), and even then, had only been collected from three localities (Cowie 1995). From then onwards, *Pomacea* spp. may have spread because these snails were mistaken for native ampullariids and were translocated to paddy fields in attempts to breed them for food – unfortunately, *Pomacea* spp. became pests that destroyed the crops instead (Khay et al. 2018). At present, *Pomacea maculata* appears to be widespread in the Tonle Sap basin.

####### Superfamily Viviparoidea Gray, 1847

**Family Viviparidae Gray, 1847**

######## Filopaludina martensi cambodjensis

Taxon classificationAnimaliaArchitaenioglossaViviparidae

(Mabille & Le Mesle, 1866)

7813EC6C-0F76-5CFB-81B5-A38518C3978E

Fig. 5E

Paludina cambodjensis Mabille & Le Mesle, 1866: 135, pl. 7, fig. 4. Type locality: “Moth-Kasa, dans les marais”.
Filopaludina (Siamopaludina) martensi cambodjensis : Brandt 1974: 28, pl. 2, fig. 24.

######### Material examined.

CIFI.MOL.033, CIFI.MOL.041, ZRC.MOL.015715, ZRC.MOL.015716, ZRC.MOL.015717, ZRC.MOL.015718, ZRC.MOL.015719, ZRC.MOL.015720, ZRC.MOL.015721, ZRC.MOL.015722, ZRC.MOL.015723, ZRC.MOL.015724, ZRC.MOL.015725, ZRC.MOL.015726, ZRC.MOL.015727, ZRC.MOL.015728, ZRC.MOL.015729, ZRC.MOL.015730, ZRC.MOL.015731, ZRC.MOL.015732, ZRC.MOL.015733.

######### Distribution and habitat.

Tonle Sap River and Lake, and surrounding watershed including rivers, irrigation ponds and paddy fields (locality no. 1, 2, 3, 5, 8, 9, 10, 11, 12, 13, 16, 17, 19, 20, 21, 22, 24, 32, 37, 38, 40, 42 and 44).

######### Remarks.

*Filopaludina martensi cambodjensis* is said to be lacking in spiral ridges compared to *Filopaludina martensi martensi* (Frauenfeld, 1865) (see Brandt 1974), but our specimens showed some variation, with some displaying ridges. The validity of the various *Filopaludina martensi* subspecies have not been investigated in detail to date. The species is sold in local markets, but did not appear to be harvested in as large quantities as *Mekongia rattei* (Crosse & Fischer, 1876), and its annual harvest at five main landing points in Kampong Chhnang Province has previously been recorded to be 68 tonnes, only ca. 1/5^th^ of the *Mekongia swainsoni* (Lea, 1856) that were obtained (Ngor et al. 2016).

######## Idiopoma umbilicata

Taxon classificationAnimaliaArchitaenioglossaViviparidae

(Lea, 1856)

563D41AB-19D0-5804-BE53-F0FE854453DA

Fig. 5F

Paludina umbilicata Lea, 1856: 109. Type locality: “Takrong River, Siam”.
Idiopoma umbilicata : Brandt 1974: 34–35, pl. 2, fig. 35.

######### Material examined.

ZRC.MOL.015734, ZRC.MOL.015735, ZRC.MOL.015736, ZRC.MOL.015737, ZRC.MOL.015738, ZRC.MOL.015739, ZRC.MOL.015740, ZRC.MOL.015741, ZRC.MOL.015742.

######### Distribution and habitat.

Tonle Sap Lake and tributaries at Kampong Chhnang, Battambang, Siem Reap and Kampong Thom Provinces, irrigation ponds and paddy fields at Banteay Meanchey and Siem Reap Provinces (locality no. 7, 13, 24, 29 and 39).

######### Remarks.

*Idiopoma umbilicata* is often found together with *Filopaludina martensi cambodjensis* and can be differentiated from the latter species by its smaller size and shouldered shells. It does not appear to be harvested for food.

######## Mekongia rattei

Taxon classificationAnimaliaArchitaenioglossaViviparidae

(Crosse & Fischer, 1876)

38F3445D-BA46-59E5-AA86-89C84DAABE13

Fig. 5G

Paludina rattei Crocsse & Fischer, 1876: 317. Type locality: “Stung Chinit, Cambodia”.
Mekongia rattei : Brandt 1974: 44–45, pl. 3, figs 51, 52.

######### Material examined.

CIFI.MOL.042, MUMNH.VIV.001, MUMNH.VIV.002, MUMNH.VIV.003, ZRC.MOL.015744, ZRC.MOL.015745, ZRC.MOL.015743.

######### Distribution and habitat.

Tonle Sap Lake; Chi Kraeng River and Sreng River in Siem Reap Province; Phumi Phsar River in Kampong Chhnang Province (locality no. 7, 8, 11, 13, 16, 20, 24, 27, 36, 37 and 39).

######### Remarks.

*Mekongia rattei* is sold in local markets surrounding the Lake. This species along with *Corbicula* spp. are commercially harvested from the Lake to be sold locally and exported abroad for human consumption and as animal feed in local poultry farms (Fig. 4B). Another record of *Mekongia* from the Tonle Sap basin and surrounding drainages is *Mekongia swainsoni* (see Brandt 1974; Ngor et al. 2018d). *Mekongia rattei* differs from *Mekongia swainsoni* by its larger size and conic shape of spire.

######## Trochotaia trochoides

Taxon classificationAnimaliaArchitaenioglossaViviparidae

(Martens, 1860)

86D6E6C2-0805-5263-A8C2-F20856134603

Fig. 5H

Paludina trochoides Martens, 1860: 12. Type locality: “Siam”.
Trochotaia trochoides : Brandt 1974: 32–33, pl. 2, figs 32, 33.

######### Material examined.

CIFI.MOL.034, ZRC.MOL.015746.

######### Distribution and habitat.

Paddy fields at Banteay Meanchey Province (locality no. 7).

######### Remarks.

We found only a few dry shells in the northwestern province of Banteay Meanchey, close to the Thai border. The species was also occasionally encountered for sale at local markets.

####### Order Littorinimorpha Golikov & Starobogatov, 1975

**Superfamily Truncatelloidea Gray, 1840**

**Family Bithyniidae Gray, 1857**

######## Bithynia siamensis goniomphalus

Taxon classificationAnimaliaLittorinimorphaBithyniidae

(Morelet, 1866)

04997351-F4C8-5251-A99F-573DD7A18CDD

Fig. 5I

Paludina goniomphalos Morelet, 1866: 167. Type locality: “Cochinchina”.
Bithynia (Digoniostoma) siamensis goniomphalus : Brandt 1974: 60, pl. 4, fig. 68.

######### Material examined.

CIFI.MOL.040, ZRC.MOL.015696, ZRC.MOL.015697, ZRC.MOL.015698, ZRC.MOL.015699, ZRC.MOL.015700, ZRC.MOL.015701.

######### Distribution and habitat.

Found at the edges of Tonle Sap River and Lake, ponds and in paddy fields at Kampong Chhnang, Banteay Meanchey, and Siem Reap Provinces (locality no. 3, 4, 8, 11, 16, 40, 42, 43, and 44).

######### Remarks.

*Bithynia siamensis goniomphalus* is of medical importance because it is an intermediate host of the zoonotic parasite, *Opisthorchis viverrini* (Poirier, 1886) (TROPMED Medical Group 1986). The role of *Bithynia siamensis goniomphalus* in the transmission of this parasite in Cambodia has not been investigated in detail, although the parasite has been recorded in freshwater fishes at the border of the Kandal-Takeo Provinces in the south (Touch et al. 2009), and cases of human infections are well-studied throughout the country (Sithithaworn et al. 2012).

######## Wattebledia siamensis

Taxon classificationAnimaliaLittorinimorphaBithyniidae

Möllendorff, 1902

969F8152-33EF-56D0-92B7-D2E68EF7906D

Fig. 5J

Wattebledia siamensis Möllendorff, 1902: 160. Type locality: “Siam”.
Wattebledia siamensis : Brandt 1974: 64–65, pl. 5, figs 78, 79.

######### Material examined.

ZRC.MOL.016325

######### Distribution and habitat.

Found among floating vegetation along the banks of the Tonle Sap River (locality no. 44).

######### Remarks.

This is the first record of the species in Cambodia, and the species is known throughout Thailand (Brandt 1974). It could probably be more widespread along with the larger congeneric species, *Wattebledia crosseana* (Wattebled, 1886), which has previously been recorded from Cambodia (Brandt 1974).

####### Order Neogastropoda Wenz, 1938

**Superfamily Buccinoidea Rafinesque, 1815**

**Family Nassariidae Iredale, 1916 (1835)**

######## Anentome cambojiensis

Taxon classificationAnimaliaNeogastropodaNassariidae

(Reeve, 1861)

544E50B2-2B5D-5B80-AB2A-ABC296C95F12

Fig. 5K

Melania cambojiensis Reeve, 1861: Melania species 468, pl. 59. Type locality: “Cambojia”.
Clea (Anentome) cambojiensis : Brandt 1974: 202.

######### Material examined.

CIFI.MOL.039, ZRC.MOL.015708, ZRC.MOL.015709, ZRC.MOL.015710, ZRC.MOL.015711, ZRC.MOL.015712, ZRC.MOL.015713, ZRC.MOL.015714.

######### Distribution and habitat.

Tonle Sap Lake; on muddy substrate (locality no. 8, 11, 12, 17 and 37).

######### Remarks.

The type specimen of *Anentome cambojiensis* was collected by Henri Mouhot (Reeve 1861) and appears to be endemic to the Tonle Sap basin. Its range may extend to eastern Thailand in Rayong (Morlet 1889), although later surveys have not recorded this species beyond Cambodia (Brandt 1974) and we did not find it in tributaries around the Lake. Brandt (1974) also did not find the species after surveying ‘the banks of Tonle Sap carefully’, but we found it to be common and widespread throughout the Lake. Locals know the species to be carnivorous or a scavenger, and we found it around dead branches that are stuck into the mud to attract shrimp.

######## Anentome helena

Taxon classificationAnimaliaNeogastropodaNassariidae

(von dem Busch in Philippi, 1847)

D1D4E590-238E-5BCE-B0CA-C689073C9385

Fig. 5L

Melania helena von dem Busch in Philippi, 1847: 170, pl. 4, fig. 4. Type locality: Java.
Clea (Anentome) helena : Brandt 1974: 201–202, pl. 15, figs 64, 65.

######### Material examined.

CIFI.MOL.035, ZRC.MOL.015702, ZRC.MOL.015703, ZRC.MOL.015704, ZRC.MOL.015705, ZRC.MOL.015706, ZRC.MOL.015707.

######### Distribution and habitat.

Tonle Sap River, Lake and surrounding tributaries, irrigation ponds and canals (locality no. 2, 7, 8, 10, 11, 13, 16, 17, 21, 22, 24, 25, 26, 34, 38, 40, 41, 42, and 44).

######### Remarks.

The species recognised as *Anentome helena* in Indo-China may in fact be a complex of three species (Strong et al. 2017), however further studies need to be conducted to determine which clade the Cambodian ones belong to. Pending systematic revision of this taxon, the name *Anentome helena* is herein used for the species in Cambodia. *Anentome helena* may be found in similar habitats as *Anentome cambojiensis* in the Lake but does not occur in as high numbers as the latter species. Beyond the Lake, it was commonly found in tributaries and other water bodies.

######## Cohort Sorbeoconcha Ponder & Lindberg, 1997

**Superfamily Cerithioidea J. Fleming, 1822**

**Family Pachychilidae Fischer & Crosse, 1892**

######### Sulcospira housei

Taxon classificationAnimaliaNeogastropodaPachychilidae

(Lea, 1856)

4B900938-94A7-58CA-ACE7-0AA1214FF893

Fig. 5M

Melania housei Lea, 1856: 144–145. Type locality: “Korat, Takrong River, Siam”.
Adamietta housei : Brandt 1974: 171–172, pl. 12, fig. 24.

########## Material examined.

CIFI.MOL.043, ZRC.MOL.015748, ZRC.MOL.015749.

########## Distribution and habitat.

Tonle Sap River, Sangkae River in Battambang Province, canal in Kampong Thom Province (locality no. 1, 2, 26, 38, 41, 43 and 44).

########## Remarks.

*Sulcospira housei* is widespread in neighbouring Thailand (Brandt 1974) and based on our study and past records (Crosse and Fischer 1876; Morlet 1889), appears to be widely distributed in Cambodia also, from around the Tonle Sap basin to the south in Kampot Province.

######### Family Thiaridae Gill, 1871 (1823)

########## Melanoides tuberculata

Taxon classificationAnimaliaNeogastropodaThiaridae

(OF Müller, 1774)

8C0EEDF4-DA90-521A-9F08-702A9D84E421

Fig. 5N

Nerita tuberculata OF Müller, 1774: 191. Type locality: “In littore Coromandel”.
Melanoides tuberculata : Brandt 1974: 164–166, pl. 12, figs 9–12.

########### Material examined.

CIFI.MOL.036, ZRC.MOL.015747, ZRC.MOL.015750, ZRC.MOL.015751, ZRC.MOL.01572.

########### Distribution and habitat.

Tonle Sap Lake and Thliem Ma-Orm River in Pursat Province (locality no. 11, 12, 20, 27 and 35).

########### Remarks.

*Melanoides tuberculata* has a global distribution but was not found to be common in the Tonle Sap basin during our surveys.

####### Subclass Heterobranchia Burmeister, 1837

**Superorder Hygrophila Férussac, 1822**

**Family Bulinidae P. Fischer & Crosse, 1880**

######## Indoplanorbis exustus

Taxon classificationAnimaliaHygrophilaBulinidae

(Deshayes, 1833)

ADD823E3-0324-547D-9213-462469C01293

Fig. 5O

Planorbis exustus Deshayes, 1833: 417, pl. 1, figs 11–13. Type locality: “Lieux man~cagieux de la cote de Malabar”.
Indoplanorbis exustus : Brandt 1974: 234–235, pl. 16, fig. 99.

######### Material examined.

ZRC.MOL.016324.

######### Distribution and habitat.

Dry shells found at a lotus pond in Banteay Meanchey Province (locality no. 5).

######### Remarks.

*Indoplanorbis exustus* has been recorded from Cambodia since the 1800s (Mabille and Le Mesle 1866; Crosse and Fischer 1876; Brandt and Temcharoen 1971), and although it was only collected from one locality during our surveys, the species is known to occur in paddy fields or shallow, ephemeral ponds across its distribution in Asia (Liu et al. 2010). The cosmopolitan species is an intermediate host of several zoonotic parasites (Liu et al. 2010).

######## Family Lymnaeidae Rafinesque, 1815

######### Radix rubiginosa

Taxon classificationAnimaliaHygrophilaLymnaeidae

(Michelin, 1831)

AFA46730-64EA-5C67-90B0-A8F8AC79C610

Fig. 5P

Lymnoeus rubiginosus Michelin, 1831: Moll. no. 22, pl. 22. Type locality: “Indes Orientales” (from Brandt 1974).
Lymnaea (Radix) auricularia rubiginosa : Brandt 1974: 229–230, pl. 16, fig. 95.

########## Material examined.

ZRC.MOL.015753, ZRC.MOL.015754.

########## Distribution and habitat.

At the edges of irrigation ponds and swampy grounds in Banteay Meanchey Province (locality no. 3).

########## Remarks.

*Radix rubiginosa* is a cosmopolitan species, widely distributed from Indo-China to Sundaland (Hubendick 1951; Brandt 1974) and is known to be a host of numerous zoonotic parasites (Brandt 1974; TROPMED Medical Group 1986). However, we only encountered the species at one locality.

## Discussion

Many of the 19^th^ to early 20^th^ century descriptions and records of freshwater molluscs from Cambodia were collected by expeditions, sponsored or led by the French, including those by the renowned traveller Henri Mouhot and the diplomat Auguste Pavie, and also by missionaries based in the country (e.g., Reeve 1861; Morelet 1865; Morlet 1889; Breure et al. 2018). The Tonle Sap Lake and surrounding watershed (e.g., Battambang) were specified as the collection locality or habitat of 33 species (Suppl. material 1: Table S1), with 20 of those species being collected from the Tonle Sap basin in our study. The family with the greatest number of species in the historical records for Cambodia, i.e., Pomatiopsidae, were previously recorded from the Mekong River only, which was not surveyed in this study. The absence of the specimens or figures in the literature prevents us from verifying the past records to check for misidentifications or taxonomic confusion, and some, for instance the *Corbicula* species, may be the same taxa that we collected. In any case, the outcome of our limited surveys recording 31 species of freshwater molluscs, including three new records for Cambodia (*Scaphula minuta*, *Novaculina siamensis*, and *Wattebledia siamensis*), appears to be the most comprehensive documentation for the Tonle Sap basin to date. In addition, the voucher specimens that we have deposited in the collection at IFReDI of the Fisheries Administration of Cambodia, represents the first known reference collection of freshwater molluscs in the country.

Our surveys have revealed that there remains much to be done in resolving the taxonomy and systematics of freshwater molluscs in Cambodia. In addition to the four species of Viviparidae that were highlighted in the Results as requiring taxonomic revision, many other taxa have not been collected or studied closely since the species were described, including the Cyrenidae, Nassariidae, and the speciose Pomatiopsidae (Suppl. material 1: Table S1). The Ampullariidae are also in need of taxonomic resolution as some records dating back to the 19^th^ century, e.g., *Ampullaria borneensis* Philippi, 1852 and *Ampullaria malabarica* Philippi, 1852, that have been synonymised with species not recognised as being distributed in Cambodia, i.e., *Pila scutata* (Mousson, 1848) and *Pila virens* (Lamarck, 1822), respectively (Cowie 2015).

Aside from the taxonomic confusion, the Ampullariidae of Tonle Sap Lake are extremely unique in terms of the high volume of production for *Pila* species being harvested from Tonle Sap Lake, compared to elsewhere in Southeast Asia, where the native ampullariids appear to be declining whilst invasive confamilial *Pomacea* species are increasing (Marwoto and Isnaningsih 2011; Ng et al. 2019, in press). *Pila virescens* is also a popular food item in neighbouring Thailand, but has extremely low genetic diversity across different populations, characteristic of species that have experienced anthropogenic translocations (Ng et al. in press). It is therefore important to conduct further research on the genetic diversity and ecology of *Pila* species within the Tonle Sap basin, especially as *Pomacea* species are to be increasing in number and spreading rapidly throughout Cambodia (Khay et al. 2018). In addition, the building of hydropower dams (i.e., reservoirs) and the change in flow of the Mekong appear to be creating new habitats that are rapidly being colonised by *Pomacea* spp. (Ngor PB, pers. obs.).

**Figure 6.**
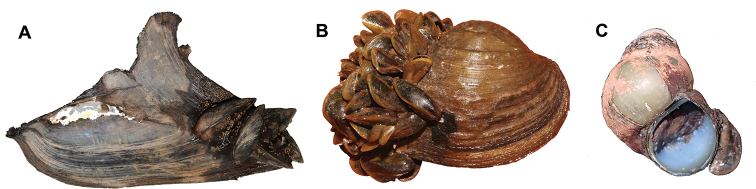
Mytilid colonies from Tonle Sap Lake, Cambodia growing on the shells of other freshwater molluscs **A***Hyriopsis bialata***B***Contradens contradens***C***Mekongia rattei*. Photographs by A Pholyotha (**A, B**) and TH Ng (**C**).

Although the mytilid mussels are assumed to be native to Cambodia, *Limnoperna fortunei* in particular, has the potential to be classified as a pest species. The mixed species colonies of *Limnoperna fortunei*, and to a lesser extent, *Sinomytilus harmandi*, have byssus threads that form dense mats, not only on hard man-made surfaces, but also on the shells of unionid mussels and gastropods (Fig. 6). Aggregations of *Limnoperna fortunei* and similarly byssate *Dreissena polymorpha* on unionids have been recorded to prevent the biological functions of the attached hosts, for e.g. *Hyriopsis bialat*a, *Hyriopsis delaportei*, and *Contradens contradens*, causing difficulties to open their valves for feeding and respiration (Karatayev et al. 2007). We observed that many unionids at edges of the Tonle Sap Lake that were particularly stagnant and covered in cyanobacteria, to be infested with the mats of mytilids (e.g., at locality no. 8, 11, and 12), especially during the dry season.

Indeed, the comtemporary Mekong River system is now at a critical point in time, facing challenges of regional development, particularly the increasing numbers of hydropower dams, which alter the timing, magnitude and frequencies of seasonal flow of the tropical flood-pulse system. Such flow alterations have been demonstrated to dampen the seasonal flood pulses (Cochrane et al. 2014) and reduce the hydro-periods as well as the open water area of the Tonle Sap Lake (Lin and Qi 2017), which supports one of the world’s largest inland fisheries (Ngor et al. 2018c). Aquatic fauna, such as freshwater fishes and molluscs, are among those that will be adversely impacted by the changes owing to the likely effects on their dispersal ability, reproductive success and rearing conditions (Ziv et al. 2012; Winemillers et al. 2016; Sabo et al. 2017; Ngor et al. 2018a, d). Combined with other anthropogenic effects in the Tonle Sap basin such as floodplain infrastructure development (Arias et al. 2019), overfishing (Ngor et al. 2018c; McCann et al. 2016), invasive species (e.g., *Pomacea* spp.), land cover change (e.g., habitat degradation) and climate change (Arias et al. 2014; Ngor et al. 2018b; Daly 2019), the future of these resources is in a precarious condition.

Our checklist is the first step toward more extensive research on freshwater molluscs in the Tonle Sap basin. It is imperative that more surveys of freshwater molluscs be conducted across different wet and dry seasons to allow for a better representation of the fauna to be captured, along with baseline data of the populations and ecology of the species to be documented. The presence of globally-invasive species like *Pomacea maculata*, and the prevalence of pest species like *Limnoperna fortunei*, which have the potential to replace and negatively impact the ecosystem and native species of the Tonle Sap basin is another major concern. In order to combat the combined pressures of invasive species, land cover change, climate change, dams along the main stem and tributaries of the Mekong River, among many other anthropogenic threats (Ngor et al. 2018b; Uk et al. 2018; Daly 2019), a multi-pronged approach is urgently required to study the biodiversity, ecology, ecosystem functioning of freshwater molluscs and other aquatic fauna in the Tonle Sap basin.

## Supplementary Material

XML Treatment for Scaphula minuta

XML Treatment for Limnoperna fortunei

XML Treatment for Sinomytilus harmandi

XML Treatment for Novaculina siamensis

XML Treatment for Corbicula

XML Treatment for Bineurus mouhotii

XML Treatment for Contradens contradens

XML Treatment for Ensidens ingallsianus

XML Treatment for Hyriopsis bialata

XML Treatment for Hyriopsis delaportei

XML Treatment for Monodontina cambodjensis

XML Treatment for Physunio micropterus

XML Treatment for Pilsbryoconcha linguaeformis

XML Treatment for Pilsbryoconcha lemeslei

XML Treatment for Scabies mandarinus

XML Treatment for Pila gracilis

XML Treatment for Pila pesmei

XML Treatment for Pila virescens

XML Treatment for Pomacea maculata

XML Treatment for Filopaludina martensi cambodjensis

XML Treatment for Idiopoma umbilicata

XML Treatment for Mekongia rattei

XML Treatment for Trochotaia trochoides

XML Treatment for Bithynia siamensis goniomphalus

XML Treatment for Wattebledia siamensis

XML Treatment for Anentome cambojiensis

XML Treatment for Anentome helena

XML Treatment for Sulcospira housei

XML Treatment for Melanoides tuberculata

XML Treatment for Indoplanorbis exustus

XML Treatment for Radix rubiginosa

## Supplementary materials

Supplementary material 1 Table S1. Historical records of freshwater molluscs from Cambodia Data type records Explanation note Historical records of freshwater molluscs from Cambodia based on literature review. https://binary.pensoft.net/file/441310This dataset is made available under the Open Database License (http://opendatacommons.org/licenses/odbl/1.0/). The Open Database License (ODbL) is a license agreement intended to allow users to freely share, modify, and use this Dataset while maintaining this same freedom for others, provided that the original source and author(s) are credited.Ting Hui Ng, Ekgachai Jeratthitikul, Chirasak Sutcharit, Samol Chhuoy, Kakada Pin, Arthit Pholyotha, Warut Siriwut, Ruttapon Srisonchai, Zeb S Hogan, Peng Bun Ngor
