# Supplementary material for: Annotated checklist of freshwater molluscs from the largest freshwater lake in Southeast Asia
Source: Zookeys. 2020 Aug 11;958:107–41. doi: 10.3897/zookeys.958.53865 (PMC7434803; doi:10.3897/zookeys.958.53865)
Supplement: Supplementary material 1 — Table S1. Historical records of freshwater molluscs from Cambodia [file zookeys-958-107-s001.pdf]

Table S1. Historical records of freshwater molluscs from Cambodia. IUCN Status follow the IUCN Red List version 2018.1—DD: Data Deficient, LC: Least Concern; NT: Near Threatened; VU: Vulnerable. Accepted names mainly follow MolluscaBase (2019) and Graf and Cummings (2019), except for species indicated by *.

Accepted name	Synonyms	IUCN Status	Source	Location in Cambodia, if specified
**BIVALVIA**				
**PTERIOMORPHA**				
**ARCIDA**				
**ARCOIDEA**				
**ARCIDAE**				
Scaphula pinna		LC	Madhyasta (2012)	"Uncertain distribution in Cambodia"
**MYTILIDA**				
**MYTILOIDEA**				
**MYTILIDAE**				
Limnoperna fortunei	*Dreisena siamensis*<br>*Modiola siamensis*<br>*Limnoperna lemeslei*<br>*Modiola cambodgensis*<br>*Modiola siamensis*<br>*Modiola* (*Limnoperna*) *siamensis*<br>*Modiola ((Limnoperna) lemeslei*<br>*Limnoperna siamensis*<br>*Limnoperna fortunei*	LC	Morelet (1866, 1875); Rochebrune (1882); Clessin (1888); Morlet (1889); Lamy (1936); Brandt (1974); Morton and Dinesen (2010)	"Battambang"<br>"…lac Tonli-Sap"<br>"Grand luc de Rham-Penh, Mekong"<br>"embouchure de la riviere de Kompong-Som"<br>"lac Tonli-Sap; Cambodge"
Sinomytilus harmandi	*Dreissena harmandi*<br>*Dreissensia crosseana*<br>*Mytilus crosseanus*<br>*Sinomytilus harmandi*	LC	Rochebrune (1882); Morlet (1884, 1889); Lamy (1936); Brandt (1974); Morton and Dinesen (2010)	“Lac de Rhom-Penh, Mekong”.<br>“Etang de Pnom-Penh (Cambodge)”<br>Tonle-Sap River…Phnom Penh, Cambodia

HETERODONTA

VENERIDA

CYRENOIDEA

CYRENIDAE

Corbicula baudoni		DD	Brandt and Temcharoen (1971)	"...in the Mekong between Pakse in Laos and Kratie in Cambodia"
Corbicula blandiana		LC	Prime (1864); Brandt and Temcharoen (1971)	Type locality: "Montes Laos, Cambodiae"<br>"...in the Mekong between Pakse in Laos and Kratie in Cambodia"
Corbicula bocourti		DD	Morlet (1889); Fischer and Dautzenberg (1904); Brandt and Temcharoen (1971)	"Grand Lac (Cambodge)"<br>"Lac Tonle-Sap, Cambodge"<br>"...in the Mekong between Pakse in Laos and Kratie in Cambodia"
Corbicula castanea	*Corbicula* aff. *sriatella*	DD	Rochebrune (1881); Fischer and Dautzenberg (1904); Brandt and Temcharoen (1971); Brandt (1974)	"...in the Mekong between Pakse in Laos and Kratie in Cambodia"
Corbicula cyreniformis	*Corbicula jullieniana*<br>*Corbicua tonkiniana*<br>*Corbicula fluminea* var. *tonkiniana*	DD	Fischer and Dautzenberg (1904); Dautzenberg and Fischer (1905); Brandt and Temcharoen (1971); Brandt (1974)	"lac Tonle-Sap et ses affluents, Cambodge"<br>"Grand Lac"<br>"...in the Mekong between Pakse in Laos and Kratie in Cambodia"<br>"…in the Mekong between Nakon Panom in Thailand and Kratie in Cambodie"
Corbicula fluminea	*Corbicula gravisi*<br>*Corbicula gryphaea*	LC	Morlet (1889); Fischer and Dautzenberg (1904); Graf and Cummings (2019)	"Haut Parsac (Cambodge)"<br>"Pursat, Cambodge"
Corbicula gubernatoria			Brandt and Temcharoen (1971)	"...in the Mekong between Pakse in Laos and Kratie in Cambodia"

Corbicula lamarckiana	*Corbicula linnaeana*	LC	Prime (1864); Brandt and Temcharoen (1971); Brandt (1974)	Type locality: "Montes Laos, Cambodiae"<br>"...in the Mekong between Pakse in Laos and Kratie in Cambodia"
Corbicula largillierti			Mabille and Le Mesle (1866); Fischer and Dautzenberg (1904)	"le grand lac du Cambodge…"<br>"Lac Tonle-Sap, Cambodge"
Corbicula leviuscula	*Corbicula laeviuscula*	DD	Brandt and Temcharoen (1971)	"...in the Mekong between Pakse in Laos and Kratie in Cambodia"
Corbicula lydigiana		DD	Fischer and Dautzenberg (1904)	"environs de Kompong-Soai, Cambodge"
Corbicula moreletiana	*Corbicula petiti*<br>*Corbicula fluminea* var. *moreletiana*	LC	Morlet (1886b); Fischer and Dautzenberg (1904); Dautzenberg and Fischer (1905); Graf and Cummings (2019)	"Grand lac du Cambodge (Tonli-Sap)"<br>"Marais sur les bords du Prec-Peam-Chilang...province de Kompong-Soai, Cambodge"<br>"lac Tonle-Sap et ses affluents, Cambodge"<br>"Cambodge: Gisement préhistorique de Somron-Seng"<br>Tonle Sap Lake, Tonle Sap River near Pursat and Phnom Penh, Battambang River
Corbicula siamensis	*Corbicula erosa*	DD	Prime (1861); Fischer and Dautzenberg (1904); Graf and Cummings (2019)	Type locality: Cambodia
Corbicula tenuis		DD	Brandt and Temcharoen (1971)	"...in the Mekong between Pakse in Laos and Kratie in Cambodia"

PALAEOHETERODONTA

UNIONIDA

UNIOIDEA

UNIONIDAE

Bineurus ellipticus	*Pseudodon ellipticus*		Conrad (1865b); Fischer (1891); Brandt and Temcharoen (1971); Graf and Cummings (2019)	Type locality: Cambodia<br>"...in the Mekong between Pakse in Laos and Kratie in Cambodia"<br>Sambor
Bineurus mouhotii	*Monocondylaea mouhotii*<br>*Monocondylaea mouhotiana*<br>*Monocondylus exilis*<br>*Pseudodon anodontinum*<br>*Pseudodon anodontinus*<br>*Pseudodon mabillei*<br>*Pseudodon manhotii*<br>*Pseudodon mouhoti*<br>*Pseudodon pierrei*<br>*Unio mouhotianus*	LC	Lea (1863, 1866); Conrad (1865a); Rochebrune (1881, 1882); Morelet (1866); Sowerby (1868); Morlet (1889); Fischer (1891); Simpson (1900); Brandt and Temcharoen (1971); Brandt (1974).	Type locality: “Laos Mountains, Cambodia, Siam”<br>“in torrentibus montanis Cambodiœ”<br>“Shigloni Breithon, Cochinchine”<br>“…entre Pnom Penh et Campot”<br>"...in the Mekong between Pakse in Laos and Kratie in Cambodia"
Bineurus thomsoni	*Pseudodon thomsoni*	DD	Morlet (1884); Fischer (1891)	Type locality: Cambodge
Chamberlainia hainesiana	*Chamberlaina duclerci*	DD	Brandt and Temcharoen (1971)	
Contradens comptus	*Unio comptus*		Deshayes and Jullien (1876); Pfeiffer et al. (2018)	
Contradens contradens	*Contradens tumidulus*<br>*Uniandra contradens fischeriana*<br>*Uniandra contradens tumidula*<br>*Uniandra contradens rustica*<br>*Unio cambodiensis*<br>*Unio cambodjensis*<br>*Unio fischerianus*<br>*Unio inornatus*<br>*Unio tumidulus*	LC	Reeve (1865); Sowerby (1866); Morlet (1883; 1889); Brandt and Temcharoen (1971); Brandt (1974)	“Grand Lac, ses affluents et les étangs du Cambodge”<br>"...in the Mekong between Pakse in Laos and Kratie in Cambodia"<br>“W-Cambodia”

Contradens crossei	*Unio crossei*<br>*Uniandra contradens crossei*		Deshayes and Jullien (1876); Brandt (1974); Jeratthitikul et al. (2019b)	Type locality: "Cambodge"<br>"Mekong and eastern affluents between Tha Uthen and Saigon"
Contradens misellus	*Trapezoideus misellus*	DD	Brandt and Temcharoen (1971)	"...in the Mekong between Pakse in Laos and Kratie in Cambodia"
Cristaria plicata	*Anodonta bellua*<br>*Dipsas bialatus*	LC	Morelet (1866); Fischer (1891); Brandt and Temcharoen (1971)	"..lacu Touli-Sap Cambogensi"
Ensidens ingallsianus	*Ensidens ingallsianus ingallsianus*<br>*Ensidens ingallsianus jaculus*<br>*Unio ingallsianus*<br>*Unio jaculus*		Crosse and Fischer (1876); Rochebrune (1882); Brandt and Temcharoen (1971); Brandt (1974); Muanta et al. (2019)	"Cambodge; province de Compong-Soai"<br>"Sombor-Sombor"<br>"...in the Mekong between Pakse in Laos and Kratie in Cambodia"<br>"Serei Sophorn River at Krong Serei Sophorn, Cambodia"
Ensidens sagittarius	*Ensidens ingallsianus dugasti*	LC	Brandt and Temcharoen (1971); Brandt (1974)	"...in the Mekong between Pakse in Laos and Kratie in Cambodia"
Harmandia somboriensis		DD	Pfeiffer et al. (2018)	
Hyriopsis bialata	*Hyriopsis bialatus*<br>*Unio delphinus*<br>*Unio delphinopterus*	LC	Mabille and Le Mesle (1866); Crosse and Fischer (1876); Fischer and Dautzenberg (1904); Dautzenberg and Fischer (1905); Suvatti (1967); Brandt and Temcharoen (1971); Brandt (1974)	"Le Grand Lac; Battambang (Cambodje)"<br>"Cambodge: Battambang"<br>"Battambang; lac Tonle-Sap et ses affluents; Mé-Khong; environs de Pnom-Penh, Cambodge"<br>"Cambodge: Gisement prehistorique de Somron-Seng"<br>"Sen River, Cambodia"<br>"...in the Mekong between Pakse in Laos and Kratie in Cambodia"

*Hyriopsis delaporte*i	*Arconaia delaporteii*<br>*Unio* (*Arconaia*) *delaportei*	LC	Crosse and Fischer (1876); Morlet (1889); Haas (1924); Brandt and Temcharoen (1971); Brandt (1974)	Type locality: “Cambodge, dans la province de Compong-Soai”<br>“Grand Lac, Cambodge”<br>“Kambodja…See Tonlé-Sap”<br>"...in the Mekong between Pakse in Laos and Kratie in Cambodia"
Hyriopsis myersiana	*Unio myersianus*	DD	Fischer (1891); Brandt (1974); Graf and Cummings (2019)	Tonle Sap
Monodontina cambodjensis	*Monocondylae cambodgenis*<br>*Monocondylea cambodjensis*<br>*Monocondylus orbicularis*<br>*Pseudodon orbicularis*<br>*Pseudodon cambodjensis*	DD	Petit de la Saussaye (1865); Mabille and Le Mesle (1866); Morelet (1866; 1875); Morlet (1889); Fischer (1891); Simpson (1900); Brandt and Temcharoen (1971); Brandt (1974); Pfeiffer and Graf (2015); Bolotov et al. (2017)	Type locality: “Battabbang, Cambodge”<br>"Battambang (Cambodje)"<br>“Battambang”<br>“Rivière du haut Pursac, Cambodge”<br>"...in the Mekong between Pakse in Laos and Kratie in Cambodia"<br>“Tonle Sap River: Pursat River”
Monodontina tumida	*Pseudodon moreleti*<br>*Pseudodon tumidus*		Fischer (1891); Mabille and Le Mesle (1866); Graf and Cummings (2019)	"Phnum-Kretch (Cambodje), torrents de la montagne"<br>Sombor, Mekong, Kampot
Monodontina vondembuschiana	*Pseudodon vondembuschianus*	LC	Fischer (1891)	
Physunio cambodiensis	*Unio cambodiensis*	LC	Morlet (1889); Brandt (1974)	
Physunio eximius		LC	IUCN (2018)	
Physunio inornatus		LC	IUCN (2018)	
Physunio micropterus	*Contradens micropterus*<br>*Physunio semialatus*<br>*Unio micropterus*<br>*Unio semialatus*	LC	Morelet (1866; 1875); Deshayes and Jullien (1876); Morlet (1889); Brandt and Temcharoen (1971); Brandt	Type locality: type locality: “in torrentibus montanis Cambodiae”<br>“Riviere de Battambang”<br>“l’arroyo de Peam-Chelang”

			(1974)	"...in the Mekong between Pakse in Laos and Kratie in Cambodia"
Physunio superbus		LC	Brandt (1974)	
Pilsbryoconcha carinifera	*Anodonta sempervivens*<br>*Pilsbryoconcha exilis*<br>*Pilsbryoconcha exilis exilis*	LC	Deshayes and Jullien (1874); Crosse and Fischer (1876); Haas (1920); Brandt and Temcharoen (1971); Brandt (1974); Graf and Cummings (2019)	" Peam Chelang, Cambodge"<br>"Arroyo de Peam Chelang…Mékong"<br>"...in the Mekong between Pakse in Laos and Kratie in Cambodia"<br>Tonle Sap
Pilsbryoconcha lemeslei	*Anodonta lemeslei*<br>*Pilsbryoconcha lemsleyi*	LC	Morelet (1875); Crosse and Fischer (1876); Fischer (1891); Simpson (1900); Brandt (1974); Graf and Cummings (2019)	"Cambodge: Battambang"<br>Tonle Sap, Battambang, Kampot
Pilsbryoconcha linguaeformis	*Anodonta linguaeformis*<br>*Pilsbryoconcha exilis linguaeformis*		Morelet (1875); Crosse and Fischer (1876); Fischer (1891); Simpson (1900); Haas (1920); Brandt (1974)	"au Cambodje, probablement dans les marécages voisins de Battambang"<br>"Marécages de Battambang…lac Tonli-Sap; étangs près de Pnom-Penh"<br>"Battambang"
Pletholophus tenuis	*Cristaria tenuis*	LC	Graf and Cummings (2019)	
Pseudodon inoscularis	*Pseudodon cumingi*<br>*Pseudodon harmandi*	LC	Fischer (1891); Brandt and Temcharoen (1971)	"...in the Mekong between Pakse in Laos and Kratie in Cambodia"
Radiatula humilis	*Scabies humilis*	DD	Brandt and Temcharoen (1971)	"...in the Mekong between Pakse in Laos and Kratie in Cambodia"
Radiatula pilata		LC	Graf and Cummings (2019)	
Scabies anceps	*Uniandra gratiosus*	DD	Graf and Cummings (2019)	

Scabies mandarinus	*Unio scobinatus*<br>*Nodularia scobinata*<br>*Unio (Nodularia) scobinatus*<br>*Indonaia crispata*<br>*Scabies scobinata*<br>*Scabies crispata*		Morelet (1875); Morlet (1889); Simpson (1900); Dautzenberg and Fischer (1905); Haas (1969); Brandt and Temcharoen (1971); Pfeiffer and Graf (2015); IUCN (2018); Pfeiffer et al. (2018); Jeratthitikul et al. (2019a)	“Battambang”<br>“Mékong, Grand Lec et ses affluents (Cambodge)”<br>“Cambodge: Somron-Seng et gisement préhistorique de Somron-Seng”<br>“Kochinchina, Kambodscha”<br>"...in the Mekong between Pakse in Laos and Kratie in Cambodia"<br>“Tonle Sap River and Mekong River in Cambodia”<br>“Serei Sophorn River, Cambodia”
Scabies ludoviciana			Brandt and Temcharoen (1971)	"...in the Mekong between Pakse in Laos and Kratie in Cambodia"
Scabies nucleus			Brandt and Temcharoen (1971)	"...in the Mekong between Pakse in Laos and Kratie in Cambodia"
Scabies phaselus			Brandt and Temcharoen (1971)	"...in the Mekong between Pakse in Laos and Kratie in Cambodia"
Sinanodonta elliptica	*Anodonta elliptica*		Fischer (1891)	
Sinanodonta lucida	*Anodonta lucida*		Fischer (1891)	
Sinanodonta woodiana	*Symphynota magnifica*<br>*Anodonta doliolum*<br>*Anodonta fusca*<br>*Anodonta magnifica*	LC	Mabille and Le Mesle (1866); Fischer (1891)	"Battambang (Cambodje)"
Trapizidens exolescens	*Trapezoideus exolescens*	LC	IUCN (2018)	
Unionetta fabagina		LC	Brandt and Temcharoen (1971); Pfeiffer et al. (2018)	"...in the Mekong between Pakse in Laos and Kratie in Cambodia"

GASTROPODA

**CAENOGASTROPODA**				
**ARCHITAENIOGLOSSA**				
**AMPULLARIOIDEA**				
**AMPULLARIIDAE**				
Pila ampullacea	*Ampullaria turbinis*	LC	Morelet (1875); Crosse and Fischer (1876); Morlet (1889); Brandt and Temcharoen (1971); Ngor et al. (2016, 2018)	"de Battambang, à l'extrémité septentrionalo du grand lac"<br>"Province de Compong"<br>"Pnom Penh (Cambodge)"
Pila gracilis	*Ampullaria gracilis*	LC	Crosse and Fischer (1876); Brandt (1974)	"Province de Compong-Soai"<br>"…on sale in markets in Kampong Chhnang province and Phnom Penh"
Pila pesmei	*Ampullaria pesmei*<br>*Ampullaria begini*	LC	Brandt and Temcharoen (1971); Vongsombath et al. (2009)	Type locality: "Phnom-Penh (Cambodge)"<br>"Le Mekong et dans tout le Cambodge"<br>"Cambodia: 1 (Mekong at Stung Treng), 2 (Mekong at Kas Lognieu and Sambor)"
Pila scutata	*Ampullaria borneensis*	LC	Morelet (1875)	Battambang
Pila virens	*Ampullaria malabarica*	LC	Morlet (1889)	Mekong (Cambodge)
Pila virescens	*Ampullaria pagoda*<br>*Ampullaria callistoma*<br>*Ampullaria polita*<br>*Ampullaria brohardi*<br>*Ampullaria polita* var. *major*	LC	Crosse and Fischer (1876); Brandt and Temcharoen (1971); Ngor et al. (2016, 2018)	"...marecages de Battambang, dans le Cambodge".<br>"Province de Compong-Soai"<br>"Pnom-Penh [Cambodge]"<br>"Cambodge: Gisement prehistorique de Somron-Seng"
Pomacea maculata			Hayes et al. (2008; 2012)	
**VIVIPAROIDEA**				
**VIVIPARIDAE**				
Anulotaia lagrandierei			Brandt and Temcharoen (1971)	"Cambodia: 1 (Mekong at Stung Treng)"

Anulotaia mekongensis		DD	Brandt (1970); Brandt and Temcharoen (1971)	Type locality: Mekong at Stung Treng near the mouth of the Sekong river<br>"Cambodia: 1 (Mekong at Stung Treng)"
Eyriesia eyriesi	*Paludina eyriesi*<br>*Paludina fischeriana*		Mabille and Le Mesle (1866); Crosse and Fischer (1876); Morlet (1889); Fischer and Dautzenberg (1904); Brandt (1974)	"...marais boises, situes aux environs de Battamban (Cambodge)"<br>"Marais des parties sud du Grand Lac, dans le voisinage du fleuve, a peu de distance de Houdong (Cambodge"<br>"Cambodge…; Battambang…"<br>"Etangs voisins du village malais de Chéran-Chomées, près Pnom Penh, Oudon (Cambodge)"<br>"Environs de Battambang; sud du lac Tonle-Sap, Krang-Chomnes, pres Pnom-Penh; Oudong, Cambodge"<br>"In Cambodia south of the Tonle Sap between Battambang and Udong"
*Filopaludina danieli**	*Paludina danieli*		Morlet (1889)	Type locality: "Etangs de Phnom Penh"
Filopaludina javanica	*Filopaludina* (*Siamopaludina*) *javanica*<br>*Filopaludina (Siamopaludina) javanica continentalis*		Brandt and Temcharoen (1971); Brandt (1974)	"Cambodia: 5 (Mekong at and opposite Kratie)"
Filopaludina martensi cambodjensis	*Paludina cambodjensis*<br>*Paludina vignesi*<br>*Paludina chalanguensis*<br>*Paludina tiranti*<br>*Filopaludina* (*Siamopaludina*) *martensi*<br>*Filopaludina (Siamopaludina) martensi cambodjensis*		Mabille and Le Mesle (1866); Morlet (1884); Deshayes and Jullien (1876); Fischer and Dautzenberg (1904); Brandt and Temcharoen (1971); Brandt (1974); Ngor et al. (2014; 2016)	Type locality: “Moth-Kasa, dans les marais”<br>“Trouvée sur le sable des flaques d'eau et du rivage de l’île Ca-C’ompung… dans les petits arroyos qui serpentent à travers les jungles du Cambodge”<br>“dans l’arroyo de Peam-Chalang”<br>“Très commune à Campot, dans les marais, les rizières, les arroyos qui communiquent avec la rivière, sur la rive gauche; à Lré Ombelle”<br>“Cambodia : Common in paddy-fields, swamps and trenches in the Mekong valley”.

				“on sale in markets in Kampong Chhnang province and Phnom Penh”.<br>Tonle Sap Lake.
*Filopaludina obscurata**	*Paludina obscurata*		Deshayes and Jullien (1876)	Type locality: "De l'arroyo de Peam-Chelang"
Filopaludina simonis	*Filopaludina* (*Siamopaludina*) *simonis*		Brandt and Temcharoen (1971)	"Cambodia: 3 (Mekong at Sandan)"
Filopaludina sumatrensis speciosa	*Filopaludina sumatrensis speciosa*		Deshayes and Jullien (1876); Brandt and Temcharoen (1971)	"Cambodia: 1 (Mekong at Stung Treng), 5 (Mekong at and opposite Kratie)"
Idiopoma dissimilis	*Paludina fulva*		Reeve (1863); Brandt (1974)	Type locality: "Cambojia"<br>“Record in Cambodia doubtful”
Idiopoma umbilicata	*Paludina ciliata*		Morlet (1889); Brandt and Temcharoen (1971); Brandt (1974)	"Mares et petits ruisseaux pres de Dei-Crochon (Cambodge)"<br>“…several localities in Cambodia”
Mekongia jullieni	*Paludina jullieni*	LC	Deshayes and Jullien (1876); Crosse and Fischer (1876); Brandt and Temcharoen (1971)	Type locality: "Trouvée dans une petite anse de l'île Ca-Lgniou, dans le sable"<br>"Ile Ca-Lgniou"<br>"Cambodia: 1 (Mekong at Stung Treng), 2 (Mekong at Kas Lognieu and Sambor), 3 (Mekong at Sandan), 5 (Mekong at and opposite Kratie)"
Mekongia lamarcki	*Paludina lamarckii*<br>*Paludina hainesiana*	DD	Deshayes and Jullien (1876); Crosse and Fischer (1876); Fischer and Dautzenberg (1904); Brandt and Temcharoen (1971)	Type locality: "l'île Ca-Lgniou"<br>"Mé-Khong a l'Ile de Ca-Lognieu, Cambodge"<br>"Cambodia: 1 (Mekong at Stung Treng), 2 (Mekong at Kas Lognieu and Sambor), 3 (Mekong at Sandan), 4 (Mekong at Samboc), 5 (Mekong at and opposite Kratie)"
*Mekongia paviei**	*Paludina paviei*		Morlet (1889)	Type locality: "Marias de Kampot (Cambodge)"

Mekongia rattei	*Paludina frauenfeldi*<br>*Paludina rattei*		Deshayes and Jullien (1876); Crosse and Fischer (1876); Morlet (1889); Fischer and Dautzenberg (1904); Brandt and Temcharoen (1971)	Type locality: "les bancs de sable du Mékong et les arroyos"<br>"Subfossile sur les bords de la riviere de Stung-Chinit"<br>"de Bassac a Siempang"<br>“Pnom Oenh; Pum Po-Bong; Cambodia”<br>"Pnom-Penh, les etangs, les petits arroyos, dans le fleuve, marais de Pum Po-Bong (Cambodge)"<br>"Pnom-Penh; Pum-Po-Bang, Cambodge…Haut-Bassac, Cmbodge...Grand Lac Tonle-Sap"<br>Tonle Sap<br>"near Phnom Penh and in the Tonle Bassac and several of its tributaries"
Mekongia sphaericula	*Paludina sphaericula*<br>*Paludina moreleti*	LC	Deshayes and Jullien (1876); Brandt and Temcharoen (1971); Brandt (1974)	Type locality: "les bancs de sable du Mékong, depuis Pnum-Pcrl), en remontant le fleuve"<br>"les bancs de sable du fleuve, au-dessus de PnumPenh"<br>"Cambodia: 1 (Mekong at Stung Treng), 2 (Mekong at Kas Lognieu and Sambor), 5 (Mekong at and opposite Kratie), 6"
Mekongia swainsoni kmeriana	*Paludomus conicus*<br>*Paludina kmeriana*<br>*Mekongia kmeriana*		Morlet (1889; 1890); Brandt and Temcharoen (1971); Brandt (1974)	Type locality: "Compong-Toul, dans le Prec Thenot (Cambodge)"<br>"Prec-Thenot a Kompong-Toul (Cambodge)"<br>"from the Mekong in North Cambodia"
*Mekongia turbinata**	*Paludina turbinata*	DD	Deshayes and Jullien (1876)	Type locality: "Trouvée dans une petite anse de l'île Ca-Lgniou, dans le sable"
Trochotaia trochoides	*Paludina trochoides*		Morlet (1889); Brandt (1974)	"Entre Pnom-Penh et Compong-Till (Cambodge)"

LITTORINIMORPHA
TRUNCATELLOIDEA

**BITHYNIDAE**				
Bithynia siamensis goniomphalus	*Bithinia goniomphalus*		Crosse and Fischer (1876); Fischer and Dautzenberg (1904)	"Grand-Lac Tonle-Sap, Mé-Khong, Cambodge"
Bithynia laevis			Fischer and Dautzenberg (1904)	"Kompong-Som; Tap-Cheang; Pnom-Penh, Cambodge"
Bythinia slriatula			Mabille and Le Mesle (1866); Fischer and Dautzenberg (1904)	"Marais et petits cours d'eau de presque tout le Cambodge"
Wattlebledia crosseana			Brandt (1974)	
**IRAVADIIDAE**				
Rehdriella parva	*Pachydrobia parva*		Morlet (1889); Fischer and Dautzenberg (1904); Brandt (1974)	"Preck.-Scholll, Mé-Khong, Cambodge"
**POMATIOPSIDAE**				
Halewisia expansa	*Manningiella expansa*	LC	Brandt (1970); Brandt and Temcharoen (1971)	"Mekong from Bandan to Sandan, N of Kratie" "Cambodia: 2 (Mekong at Kas Lognieu and Sambor), 3 (Mekong at Sandan), 4 (Mekong at Samboc)"
Hubendickia sulcata	*Hubendickia spiralis*	LC	Brandt and Temcharoen (1971); Brandt (1974)	"Cambodia: 2 (Mekong at Kas Lognieu and Sambor), 4 (Mekong at Samboc)" "Mekong between Kemmarath and Sambor in N Cambodia"
Hydrorissoia cambodiensis		DD	Brandt (1970); Brandt and Temcharoen (1971)	Type locality: Mekong at Sambor, North Cambodia

Hydrorissoia elegans		DD	Brandt and Temcharoen (1971)	"Cambodia: 2 (Mekong at Kas Lognieu and Sambor), 4 (Mekong at Samboc)"
Hydrorissoia gracilis		LC	Brandt and Temcharoen (1971)	"Cambodia: 2 (Mekong at Kas Lognieu and Sambor), 3 (Mekong at Sandan)"
Hydrorissoia hospitalis		LC	Brandt and Temcharoen (1971)	"Cambodia: 2 (Mekong at Kas Lognieu and Sambor), 4 (Mekong at Samboc)"
Hydrorissoia paviei		LC	Brandt (1970)	Type locality: Mekong at Sambor, North Cambodia
Hydrorissoia waltoni			Brandt and Temcharoen (1971)	Type locality: Mekong at Sambor in North Cambodia
Jullienia acuta		DD	Poirier (1881); Fischer and Dautzenberg (1904); Brandt and Temcharoen (1971)	Type locality: Cambodia<br>"Mé-Khong, Cambodge…a Sambor"<br>"Cambodia: 2 (Mekong at Kas Lognieu and Sambor), 3 (Mekong at Sandan)"
Jullienia costata	*Wykoffia costata*	VU	Poirier (1881); Fischer and Dautzenberg (1904); Brandt and Temcharoen (1971)	Type locality: Cambodia<br>"Rapides de Sambor, Mé-Khong, Cambodge"
Jullienia flava	*Melania flava*<br>*Lithoglyphus* (*Jullienia*) *flavus*	VU	Deshayes and Jullien (1876); Crosse and Fischer (1876); Fischer and Dautzenberg (1904)	Type locality: "les rivages sablonneux du Mékong, sur divers points au-dessus de Koko; elle est commune"<br>"Le Mé-Khong, au -dessus de Koko; rapides de Prec-Ompil, Cambodge"
Jullienia harmandi		LC	Poirier (1881); Fischer and Dautzenberg (1904); Brandt and Temcharoen (1971)	"Rapides de Sambor, Mé-Khong, Cambodge"<br>"Cambodia: 2 (Mekong at Kas Lognieu and Sambor)"
Jullienia microsculpta		DD	Brandt (1970); Brandt and Temcharoen (1971)	Type locality: Mekong at Sambor<br>"Cambodia: 2 (Mekong at Kas Lognieu and Sambor), 3 (Mekong at Sandan)"

Jullienia nodulosa		DD	Poirier (1881); Fischer and Dautzenberg (1904)	Type locality: Cambodia<br>"Mé-Khong, Cambodge"
Jullienia nucula			Brandt and Temcharoen (1971)	Cambodia: 2 (Mekong at Kas Lognieu and Sambor)
Jullienia poirieri			Brandt (1970); Brandt and Temcharoen (1971)	Type locality: Mekong at Sambor<br>"Cambodia: 2 (Mekong at Kas Lognieu and Sambor), 3 (Mekong at Sandan)"
Jullienia tricostata	*Lacunopsis tricostatus*<br>*Wykoffia tricostata*	DD	Deshayes and Jullien (1876); Brandt and Temcharoen (1971)	Type locality: "les pierres aux rapides de Tio-Compih"<br>"Rapides de Prec-Ompil, Cambodge"<br>"Cambodia: 2 (Mekong at Kas Lognieu and Sambor), 3 (Mekong at Sandan), 4 (Mekong at Samboc), 5 (Mekong at and opposite Kratie)"
Karelainia davisi	*Paraprososthenia davisi*	LC	Brandt and Temcharoen (1971)	"Cambodia: 2 (Mekong at Kas Lognieu and Sambor)"
Karelainia hydrorissoidea	*Paraprososthenia hydrorissoidea*	NT	Brandt and Temcharoen (1971)	"Cambodia: 2 (Mekong at Kas Lognieu and Sambor)"
Karelainia vivoni	*Paraprososthenia vivonai*	LC	Brandt and Temcharoen (1971)	"Cambodia: 2 (Mekong at Kas Lognieu and Sambor)"
Lacunopsis concava		LC	Brandt and Temcharoen (1971)	"Cambodia: 3 (Mekong at Sandan)"
Lacunopsis conica		LC	Brandt and Temcharoen (1971)	"Cambodia: 2 (Mekong at Kas Lognieu and Sambor), 3 (Mekong at Sandan)"
Lacunopsis fischerpiettei		NT	Brandt and Temcharoen (1971)	"Cambodia: 2 (Mekong at Kas Lognieu and Sambor), 3 (Mekong at Sandan)"

Lacunopsis globosa	VU	Poirier (1881); Fischer and Dautzenberg (1904); Brandt and Temcharoen (1971)	Type locality: Cambodia<br>"Mé-Khong, Camodge…Mé-Khong Sambor"<br>"Cambodia: 2 (Mekong at Kas Lognieu and Sambor), 5 (Mekong at and opposite Kratie)"
Lacunopsis harmandi	LC	Poirier (1881); Fischer and Dautzenberg (1904); Brandt and Temcharoen (1971)	Type locality: Cambodia<br>"Mé-Khong a Sambor"<br>"Cambodia: 2 (Mekong at Kas Lognieu and Sambor), 5 (Mekong at and opposite Kratie)"
Lacunopsis jullieni	LC	Deshayes and Jullien (1876); Fischer and Dautzenberg (1904); Brandt and Temcharoen (1971)	"Mé-Khong a l'ile Ca-Lognieu, Cambodge…Mé-Khong, a Sambor"<br>"Cambodia: Known from the Mekong at the island of Kas Lognieu and Sambor"
Lacunopsis monodonta	DD	Deshayes and Jullien (1876); Fischer and Dautzenberg (1904); Brandt and Temcharoen (1971)	Type locality: Mekong River, Cambodia<br>"Mé-Khong, Ile Ca-Lognieu, Cambodge…Mé-Khong a Sambor"<br>"Cambodia: 2 (Mekong at Kas Lognieu and Sambor), 5 (Mekong at and opposite Kratie)"
Lacunopsis sphaerica	DD	Brandt and Temcharoen (1971)	"Cambodia: 2 (Mekong at Kas Lognieu and Sambor), 3 (Mekong at Sandan), 5 (Mekong at and opposite Kratie)"
Lacunopsis ventricosa	DD	Poirier (1881); Fischer and Dautzenberg (1904); Brandt and Temcharoen (1971)	Type locality: Cambodia<br>"Mé-Khong, Cambodge…Rapides du Mé-Khong, a Sambor"<br>"Cambodia: 2 (Mekong at Kas Lognieu and Sambor)
Manningiella cambodiensis		Brandt (1970); Brandt and Temcharoen (1971)	Type locality: Mekong river at Sambor, Cambodia<br>"Cambodia: 2 (Mekong at Kas Lognieu and Sambor), 3 (Mekong at Sandan), 4 (Mekong at Samboc)"

Neoprososthenia hanseni	*Paraprososthenia hanseni*	LC	Brandt (1970); Brandt and Temcharoen (1971)	Type locality: Mekong at Sambor in Cambodia<br>"Cambodia: 2 (Mekong at Kas Lognieu and Sambor)"
Neoprososthenia ijimai	*Paraprososthenia iijimai*	LC	Brandt (1970); Brandt and Temcharoen (1971)	"Known from the Mekong between the type locality (Bandan, East Thailand) and Sambor in North Cambodia"<br>"Cambodia: 2 (Mekong at Kas Lognieu and Sambor)"
Neoprososthenia levayi			Brandt and Temcharoen (1971)	"Cambodia: 2 (Mekong at Kas Lognieu and Sambor)"
Neoprososthenia poirieri	*Pachydrobia poirieri*	LC	Brandt (1970); Brandt and Temcharoen (1971)	Type locality: Mekong at Sambor<br>"Cambodia: 2 (Mekong at Kas Lognieu and Sambor), 3 (Mekong at Sandan)"
Neotricula aperta		LC	Attwood et al. (2004)	Xe Kong river, Stung Treng, Cambodia
Pachydrobia bavayi		LC	Brandt (1970); Brandt and Temcharoen (1971)	Type locality: Mekong at Kratie in Cambodia
Pachydrobia mcmulleni			Brandt (1970); Brandt and Temcharoen (1971)	Type locality: "Mekong branm at Phung Krangdtam about 16 km SW of Stung Treng. Tihis is the western branch of the Mekong at Kas (= island) Satay"<br>"Cambodia: 1 (Mekong at Stung Treng)"
Pachydrobia paradoxa		LC	Crosse and Fischer (1876); Fischer and Dautzenberg (1904); Brandt and Temcharoen (1971)	Type locality: "Cambodge, dans les bancs de sable du Mekong"<br>Mé-Khong, Sambor, Cambodge"<br>"Mekong in Cambodia: 2 (Mekong at Kas Lognieu and Sambor), 5 (Mekong at and opposite Kratie), 6"
Pachydrobia spinosa		LC	Poirier (1881); Fischer and Dautzenberg (1904); Brandt	"Ille Denn, Bassac"

		(1974)	
Pachydrobia variabilis	LC	Poirier (1881); Brandt (1974)	"Mekong from Bandan to Sandan in Cambodia"
Pachydrobiella brevis	NT	Brandt and Temcharoen (1971)	"Cambodia: 2 (Mekong at Kas Lognieu and Sambor)"
Paraprososthenia acicula	NT	Brandt (1970); Brandt and Temcharoen (1971)	Type locality: Mekong at Sambor in Cambodia "Cambodia: 2 (Mekong at Kas Lognieu and Sambor)"
Paraprososthenia adami	LC	Brandt (1970); Brandt and Temcharoen (1971)	Type locality: Mekong at Sambor in Cambodia "Cambodia: 2 (Mekong at Kas Lognieu and Sambor)"
Paraprososthenia bollingi	NT	Brandt (1970); Brandt and Temcharoen (1971)	Type locality: Mekong at Sambor in Cambodia "Cambodia: 2 (Mekong at Kas Lognieu and Sambor)"
Paraprososthenia fischerpiettei	LC	Brandt (1970); Brandt and Temcharoen (1971)	Type locality: Mekong at Sambor "Cambodia: 2 (Mekong at Kas Lognieu and Sambor)"
Saduniella planispira		Brandt (1970); Brandt and Temcharoen (1971)	Type locality: Mekong at Sambor, North Cambodia
**STENOTHYRIDAE**			
Stenothyra basisculpta	LC	Brandt (1970); Brandt and Temcharoen (1971)	"Mekong between Ban Khum, N of Bandan, and Kratie in North Cambodia" "Cambodia: 5 (Mekong at and opposite Kratie)"

Stenothyra cambodiensis		LC	Brandt and Temcharoen (1971)	Type locality: Mekong at Sambor, North Cambodia<br>Mekong between Bandan and Kratie
Stenothyra hybocystoides		LC	Brandt and Temcharoen (1971)	Cambodia: 2 (Mekong at Kas Lognieu and Sambor), 5 (Mekong at and opposite Kratie)
Stenothyra mcmulleni		DD	Brandt (1970); Brandt and Temcharoen (1971)	"Mekong between Ban Khum and Kratie"<br>"Cambodia: 5 (Mekong at and opposite Kratie)"
Stenothyra roseni		DD	Brandt and Temcharoen (1971)	"Cambodia: 2 (Mekong at Kas Lognieu and Sambor)"
Stenothyra schuetti		LC	Brandt and Temcharoen (1971)	"Cambodia: 2 (Mekong at Kas Lognieu and Sambor)"
**NEOGASTROPODA**				
**BUCCINOIDEA**				
**NASSARIIDAE**				
Anentome bizonata	*Canidia bizonata*		Deshayes and Jullien (1876)	Type locality: "l'ile de Ca-Lgniou"
Anentome cambojiensis	*Clea cambodjensis*<br>*Clea* (*Anentome*) *cambojiensis*<br>*Hemisinus cambodjensis*<br>*Melania cambojiensis*<br>*Semisinus cambodgensis*		Reeve (1861); Mabille and Le Mesle (1866); Morlet (1899); Brandt and Temcharoen (1971); Brandt (1974)	Type locality: "Cambojia" ["Tonle Sap, Cambodia N of Battambang" from Brandt, 1974])<br>"Le Grand Lac et les environs de Battambang (Cambodje)"<br>"Grand lac (Cambodge)"<br>"Tonle Sap and from Battambang in Cambodia"
Anentome fusca	*Canidia fusca*<br>*Clea fusca*		Adams (1861); Brandt and Temcharoen (1971)	Type locality: "Camboja"<br>"Mekong at Kas Lognieu and Sambor"
Anentome helena	*Clea fusiformis*<br>*Clea helena*<br>*Clea* (*Anentome*) *fusiformis*		Mabille and Le Mesle (1866); Deshayes and Jullien (1876); Brandt and Temcharoen (1971)	"Les marais du Grand Lac, aux environs de Houdong (Cambodje)"<br>"l'ile de Ca-Lgniou (Cambodge)"

	Clea (*Anentome*) *helena*<br>*Hemisinus baudonianus*			“Cambodia: 2 (Mekong at Kas Lognieu and Sambor), 3 (Mekong at Sandan), 5 (Mekong at and opposite Kratie)”, “Cambodia: 2 (Mekong at Kas Lognieu and Sambor)”
Anentome jullieni	*Canidia jullieni*<br>*Canidia broti*<br>*Clea jullieni*	DD	Deshayes and Jullien (1876); Brandt and Temcharoen (1971)	Type locality: “l’ile de Ca-Lgniou"<br>"Mekong at Kas Lognieu and Sambor"
Anentome paviei	*Canidia paviei*<br>*Clea paviei*		Morlet (1886a); Brandt and Temcharoen (1971)	Type locality: "Les rapides de Sambor, Mekong, Cambodge"<br>"Mekong at Kas Lognieu and Sambor"
Anentome scalarina	*Canidia scalarina*<br>*Clea scalarina*		Deshayes and Jullien (1876); Brandt and Temcharoen (1971)	Type locality: “l’ile de Ca-Lgniou"<br>"Mekong at Kas Lognieu and Sambor" and "Mekong at and opposite Kratie"

SORBEONCONCHA

CERITHIOIDEA

PACHYCHILIDAE

Brotia jullieni	*Melania jullieni*<br>*Brotia costula jullieni*	DD	Deshayes and Jullien (1876); Brandt and Temcharoen (1971)	Type locality: "Cambodia, raides de Tio Compih, au-dessous de Somboc"<br>"Cambodia: 5 (Mekong at and opposite Kratie)"
Sulcospira housei	*Melania housei*<br>*Melania schomburgki*<br>*Adamietta housei*	LC	Crosse and Fischer (1876); Morlet (1889); Brandt, 1974)	"Dans les torrents de la province de Compong-Soai”.<br>“Pnom-Penh et ses environs, rivière de Mongkol-Borey, ruisseau se jetant dans le Prec-Thenot, sur la rive droite, dans les environs de Kompong-Toul, Kampot (Cambodge)”

**THIARIDAE**				
Melanoides tuberculata	*Melania tuberculata*<br>*Melania virgulata*	LC	Morlet (1889); Brandt and Temcharoen (1971)	“Dans le Streng-Dontri à Kassan-Pno (Cambodge), Kompong-Kal, rivière de Battambang (Siam) [now Cambodia]”<br>“…at many places in the Mekong River”
Mieniplotia scabra	*Melania spinulosa*	LC	Morlet (1899)	"Dans le Streng-Dontri, à Kassan-Pno (Cambodge)"
**HETEROBRANCHIA**				
**HYGROPHILA**				
**LYMNAEOIDEA**				
**BULINIDAE**				
Indoplanorbis exustus	*Planorbis circumspissus*	LC	Mabille and Le Mesle (1866); Crosse and Fischer (1876); Brandt and Temcharoen (1971)	"Moth-Kasa (Cambodje)"
**LYMNAEIDAE**				
Radix rubiginosa	*Limnaea crosseana*<br>*Lymnaea auricularia rubiginosa*<br>*Lymnaea* (*Radix*) *auricularia rubiginosa*	LC	Mabille and Le Mesle (1866); Hubendick (1951); Brandt and Temcharoen (1971)	"Moth-Kasa (Cambodje)"<br>“Common in ponds and marshes bordering the Mekong”

References

Adams H (1861). Descriptions of some new genera and species of shells from the collection of Hugh Cuming, Esq. Proceedings of the Zoological Society of London 1861 (3): 383–385.

Attwood SW, Campbell I, Upatham ES, Rollinson D (2004) Schistosomes in the Xe Kong River of Cambodia: the detection of *Schistosoma mekongi* in a natural population of snails and observations on the intermediate host's distribution. Annals of Tropical Medicine and Parasitology 98: 221–230. https://doi.org/10.1179/000349804225003325

Bolotov IN, Vikhrev IV, Kondakov AV, Konopleva ES, Gofarov MY, Aksenova OV, Tumpeesuwan S (2017). New taxa of freshwater mussels (Unionidae) from a species-rich but overlooked evolutionary hotspot in Southeast Asia. Scientific Reports 7: 11573. https://doi.org/ doi:10.1038/s41598-017-11957-9

Brandt RAM (1970) New freshwater gastropods from the Mekong. Archiv für Molluskenkunde 100: 183–205.

Brandt RAM (1974) The non-marine aquatic Mollusca of Thailand. Archiv für Molluskenkunde 105: 1–423.

Brandt RAM, Temcharoen P (1971) The molluscan fauna of the Mekong at the foci of Schistosomiasis in South Laos and Cambodia. Archiv für Molluskenkunde 101: 111–140.

Conrad TA (1865a) Remarks on the genera *Monocondylœa*, D'Orb., and *Pseudodon* Gould, with a synopsis of the latter. American Journal of Conchology 1: 232–233.

Conrad TA (1865b) Description of a new species of Pseudodon. American Journal of Conchology 1: 352.

Crosse H, Fischer P (1876) Mollusques fluviatiles, recueillis au Cambodge par la mission scientifique Francaise de 1873. Journal de Conchyliologie 16 (4): 313–342.

Dautzenberg P, Fischer H (1905) Liste des mollusques récoltés par M. H. Mansuy en Indo-Chine et au Yunnan et description d'espèces nouvelles. Journal de Conchyliologie 53: 343–471.

Deshayes GP, Jullien J (1876) Mémoire sur les mollusques nouveaux du Cambodge. Nouvelles annales du Muséum d'histoire naturelle 10: 115–162.

Fischer P (1891) Catalogue et Distribution Géographique des Mollusques Terrestres, Fluviatiles and Marins d'une Partie de l'Indo-Chine (Siam, Laos, Cambodge, Cochinchine, Annam, Tonkin). Autun, Imprimerie Dejussieu Pere et Fils. 192 pp.

Fischer H, Dautzenberg P (1904) Catalogue des mollusques terrestres et fluviatiles de l'Indo-Chine orientale cites jusqu' à ce jour. In: Mission Pavie. Etudes divers, III, pp. 1–61.

Graf DL, Cummings KS (2019) The Freshwater Mussels (Unionoida) of the World (and other less consequential bivalves). MUSSEL Project Web Site, http://www.mussel-project.net/. [accessed 1 November 2019].

Haas F (1969) Superfamilia Unionacea. Das Tierreich 88: 1–663.

Haas F (1924) Beiträge zu einer monographie der asiatischen Unioniden. Abhandlungen der Senckenbergischen Naturforschenden Gesellschaft 38: 129–203.

Haas F (1920) Die Unioniden. In: Küster HC (Ed.) Systematisches Conchylien-Cabinet von Martini und Chemnitz 9: 293–303.

Hayes KA, Joshi RC, Thiengo SC, Cowie RH (2008) Out of South America: multiple origins of non-native apple snails in Asia. Diversity and Distributions 14: 701–712. https://doi.org/10.1111/j.1472-4642.2008.00483.x

Hayes KA, Cowie RH, Thiengo SC, Strong EE (2012) Comparing apples with apples: clarifying the identities of two highly invasive Neotropical Ampullariidae (Caenogastropoda). Zoological Journal of the Linnean Society 166: 723–753. https://doi.org/10.1111/j.1096-3642.2012.00867.x

Hubendick B (1951) Recent Lymnaeidae. Kungl. Svenska Vetenskapsakademiens Handlingar 3: 1–223.

IUCN (2018) The IUCN Red List of Threatened Species. Version 2018-2. http://www.iucnredlist.org. [accessed 22 February 2019].

Jeratthitikul E, Sutcharit C, Prasankok P (2019a) Molecular phylogeny of the Indochinese freshwater mussel genus *Scabies* Haas, 1911 (Bivalvia: Unionidae). Tropical Natural History 19 (1): 21–36.

Jeratthitikul E, Phuangphong S, Sutcharit C, Prasankok P, Kongim B, Panha S (2019b) Integrative taxonomy reveals phenotypic plasticity in the freshwater mussel *Contradens contradens* (Bivalvia: Unionidae) in Thailand, with a description of a new species. Systematics and Biodiversity 17: 134–147. https://doi.org/10.1080/14772000.2018.1554607

(Küster HC and) Clessin S (1840–90) Die Familie der Mytilidae. In Abbildungen nach der Natur mit Beschreibungen. Begonnen von Dr. H. Küster, fortgeführt und beendigt von S. Clessin. In: Martini FHW, Chemnitz JH (Eds) Systematisches Conchylien Cabinet (2nd ed), Nürnberg, 8(3), 1–170, col. pl. 1–36.

Lamy E (1936) Revision des Mytilidae vivants du Museum National D'Histoire Naturelle de Paris. Journal de Conchyliologie 80: 308–362.

Lea I (1863) Description of a new species of *Unio* and a *Monocondylœa*. Proceedings of the Academy of Natural Sciences of Philadelphia 15: 190.

Lea I (1866) New Unionidæ, Melanidæ, etc., chiefly of the United States. Journal of the Academy of Natural Sciences 6: 5–65.

Mabille J, Le Mesle G (1866) Observations sur la faune malacologique de la Cochinchine et du Cambodje, comprenant la description des espèces nouvelles. Journal de Conchyliologie 14 (2): 117–138.

Madhyastha A (2012) *Scaphula pinna*. The IUCN Red List of Threatened Species 2012: e.T171815A1332569. http://dx.doi.org/10.2305/IUCN.UK.2012-1.RLTS.T171815A1332569.en. [accessed 22 February 2019].

Morelet A (1866) Diagnoses de coquilles nouvelles de l'Indo-Chine. Revue et Magasin de Zoologie Pure et Appliquee 18: 165–168.

Morelet A (1875) Séries Conchyliologiques Comprenant L'énumération de Mollusques Terretres et Fluviatiles Recueillis Pendant le Cours de Différents Voyages, Ainsi que la Description de Plusieurs Espèces Nouvelles. F. Savy, Libraire-Éditeur, Paris. 368 pp.

Morlet L (1883) Description d'espèces nouvelles de coquilles recueillies, par M. Pavie, au Cambodge. Journal de Conchyliologie 31: 104–110.

Morlet L (1884) Description d'especes nouvelles de coquilles, recueillies par M. Pavie, au Cambodge. Journal de Conchyliologie 25: 386–403.

Morlet L (1886a) Diagnoses Molluscorum novorum Cambodgiae. Journal de Conchyliologie 34: 74–75.

Morlet L (1886b) Liste des coquilles recueillies, au Tonkin, par M. Jourdy, chef d'escadron d'artillerie, et description d'especes nouvelles. Journal de Conchyliologie 34: 257–295.

Morlet L (1889) Catalogue des coquilles recueillies, par M. Pavie, dans le Cambodge et le Royaume de Siam, et description d'espèces nouvelles. Journal de Conchyliologie 37 (3): 121–200.

Morton B, Dinesen GE (2010) Colonization of Asian freshwaters by the Mytilidae (Bivalvia): a comparison of *Sinomytilus harmandi* from the Tonle-Sap River, Phnom Penh, Cambodia, with *Limnoperna fortunei*. Molluscan Research 30 (2): 57–72.

Muanta S, Jeratthitikul E, Panha S, Prasankok P (2019) Phylogeography of the freshwater bivalve genus *Ensidens* (Unionidae) in Thailand. Journal of Molluscan Studies 85 (2): 224–231. https://doi.org/10.1093/mollus/eyz013

Ngor PB, Chhuon K, Prak LH (2016) Cambodia completes first pilot study of Tonle Sap mollusc fishery. Catch and Culture 22: 4–13.

Ngor PB, Chhuon K, Prak LH (2014) Cambodia launches pilot study to assess Tonle Sap mollusc fishery. Catch and Culture 20: 8–13.

Petit de la Saussaye S (1865) Note sur le genre Monocondylea de d'Orbigny et description d'une espece nouvelle. Journal de Conchyliologie 13: 15–19.

Pfeiffer JM III, Graf DL (2015) Evolution of asymmetrical glochidia. Zoological Journal of the Linnean Society 175 (2): 307–318. https://doi.org/10.1111/zoj.12282

Pfeiffer JM, Graf DL, Cummings KS, Page LM (2018) Molecular phylogeny and taxonomic revision of two enigmatic freshwater mussel genera (Bivalvia: Unionidae incertae sedis: *Harmandia* and *Unionetta*) reveals a diverse clade

of Southeast Asian Parreysiinae. Journal of Molluscan Studies 58: 403–413. https://doi.org/ 10.1093/mollus/eyy028

Poirier J (1881) Description de quelques especes nouvelles du Cambodge aux genres *Lacunopsis*, *Jullienia* et *Pachydrobia*. Journal de Conchyliologie 29: 6–19.

Prime T (1861) Descriptions of new species of *Cyrena*, *Corbicula* and *Sphaerium*. Proceedings of the Academy of Natural Sciences of Philadelphia 1861: 125–128.

Prime T (1864) Notes on the species of the family Corbiculadae, with figures. Annals of the Lyceum of Natural History of New York 8: 57–92, 213–237, 414–418.

Reeve LA (1861) Monograph of the genus *Melania*. Conchologia Iconica XII: 1–251.

Reeve LA (1863) Monograph of the genus *Paludina*. Conchologia Iconica XIV: 1–48.

Reeve LA (1865) *Unio inornatus*. Conchologia Iconica 16: Pl. XXIX, fig. 147.

Rochebrune A-TD (1881) Documents sur la faune malacologique de la Cochinchine et du Cambodge. Bulletin de la Société Philomathique de Paris Series 7 Volume 6: 35–74.

Rochebrune A-TD (1882) Supplement aux documents sur la faune malacologique de la Cochinchine et du Cambodge. Bulletin de la Société Philomathique de Paris Series 7 Volume 6: 99–118.

Simpson CT (1900) Synopsis of the Naiades : or pearly fresh-water mussels. Proceedings of the United States National Museum XXII: 501–1044.

Sowerby GB II (1868*) Unio mouhotianus*. Conchologia Iconica 16: Pl. XCII, fig. 503.

Sowerby GB II (1866) *Unio cambojensis*. Conchologica Iconica 16: Pl. XLII, fig. 231.

Suvatti C (1967) Mollusca. In Fauna of Thailand 2nd edition. Applied Scientific Research Corporation of Thailand, Bangkok, pp. 32–126.

Vongsombath C, Pham AD, Nguyen TML, Kunpradid T, Davison SP, Peerapornpisal Y, Sok K, Meng M (2009) Report on the 2007 Biomonitoring Survey of the Lower Mekong River and Selected Tributaries. MRC Technical Paper No. 23. Mekong River Commission, Vientiane. 75 pp.
